# Supplementary material for: Is the growth of the child of a smoking mother influenced by the father's prenatal exposure to tobacco? A hypothesis generating longitudinal study
Source: BMJ Open. 2014 Jul 11;4(7):e005030. doi: 10.1136/bmjopen-2014-005030 (PMC4120379; doi:10.1136/bmjopen-2014-005030)
Supplement: Author's manuscript [file bmjopen-2014-005030.draft_revisions.pdf]

Is the growth of the child of a smoking mother influenced by the father's prenatal exposure to tobacco? A hypothesis generating longitudinal study

Journal:	*BMJ Open*
Manuscript ID:	bmjopen-2014-005030
Article Type:	Research
Date Submitted by the Author:	10-Feb-2014
Complete List of Authors:	Pembrey, Marcus; University of Bristol, Social and Community Medicine<br>Northstone, Kate; University of Bristol, Centre for Child and Adolescent Health<br>Gregory, Steven; University of Bristol, Social and Community Medicine<br>Miller, Laura; University of Bristol, Social and Community Medicine<br>Golding, Jean; University of Bristol, Centre for Child and Adolescent Health
**Primary Subject Heading**:	Epidemiology
Secondary Subject Heading:	Public health
Keywords:	Transgenerational effects, ALSPAC, grandmothers' prenatal smoking, birth head circumference

SCHOLARONE™ Manuscripts

For peer review only

Is the growth of the child of a smoking mother influenced by the father's prenatal exposure to tobacco? A hypothesis generating longitudinal study

Marcus Pembrey[abc], Kate Northstone[a], Steven Gregory[ab], Laura L Miller[a], Jean Golding[ab]

[a]School of Social and Community Medicine, [b]Centre for Child and Adolescent Health, University of Bristol, Bristol and [c] Institute of Child Health, University College London, London, UK

Key Words: ALSPAC, transgenerational effects, grandmothers' prenatal smoking, birth head circumference, IQ

Correspondence to:

Professor Jean Golding,
Centre for Child and Adolescent Health,
School of Social and Community Medicine,
Barley House,
Oakfield Grove
Bristol BS8 2BN,
UK

Email: jean.golding@bristol.ac.uk
Telephone: +44 (0)117 3310198 Fax: +44 (0)117 3313303

5.2.14 **Word Count: 3432**

For peer review only

Abstract

Objectives: Transgenerational effects of different environmental exposures is becoming of major interest, with rodent experiments focusing on epigenetic mechanisms. Previously we have shown that if the study mother is a non-smoker, there is increased mean birth weight, length and body mass index [BMI] in her sons if she herself had been exposed prenatally to her mother's smoking. The aim of this study was to determine whether the prenatal smoke exposure of either parent influenced the growth of the fetus of a smoking woman, and whether any effects were dependent on the fetal sex.

Design: Population based pre-birth cohort study.

Setting: Avon Longitudinal Study of Parents and Children.

Participants: Participants were residents of a geographic area with expected date of delivery between April 1991 and December 1992. Among pregnancies of mothers who smoked during pregnancy, data were available concerning maternal and paternal prenatal exposures to their own mother smoking for 3502 and 2354 respectively

Primary and secondary outcome measures:, Birth weight, length, BMI and head circumference.

Results: After controlling for confounders, there were no associations with birth weight, length or BMI. There was a strong adjusted association of birth head circumference among boys whose fathers had been exposed prenatally [mean difference -0.35cm; 95%CI -0.57,-0.14; P=0.001]. There was no such association with girls (interaction: P=0.006). Similar associations were found when primiparae and multiparae were analysed separately. In order to determine whether this was reflected in child development, we examined the relationships with IQ; we found that the boys born to exposed fathers had lower IQ scores on average, and that this was particularly due to the verbal component [mean difference in verbal IQ: -3.65; 95%CI -6.60,-0.70 points].

For peer review only

Conclusions: Head size differences concerning paternal fetal exposure to smoking were unexpected and, as such, should be regarded as hypothesis generating.

For peer review only

Strengths and limitations of this study

- This study is the first to examine the sex-specific fetal effects of parental prenatal exposure to cigarette smoking when the mother herself smoked during pregnancy.
- Data were collected on a population sample who completed questionnaires blind to the study hypotheses.
- Birth measurements were undertaken using trained staff with repeated validation.
- A variety of sensitivity analyses were undertaken, including separate analyses of primiparae and multiparae, as well as of follow-up of the offspring to determine whether the decrement in birth head circumference was reflected in childhood measurement of IQ. All were in accord with the initial finding.
- The limitation of the study is the failure to obtain comparable data to confirm or negate the study findings.

For peer review only

INTRODUCTION

Fetal programming via the mother's nutrition and other aspects of her environment is well-recognised as a contributor to adult morbidity and mortality[1] and some of these enduring effects are likely to be mediated by epigenetic mechanisms.[2,3] Studies have shown specific DNA methylation patterns in children whose mothers had smoked during pregnancy.[4-7] However, there have been few pre-conceptional transgenerational studies relating the fetal environment of either parent to the birth outcomes of their own children.

In an earlier study of non-smoking mothers, we found an increase in the birth weight and birth body mass index [BMI] of her sons if she had been exposed *in utero* to her own mothers' smoking, but there was no such effect if the study father had been exposed *in utero*.[8] This lack of paternal influence from his own intrauterine exposure was not unexpected. Indeed, it has been proposed that the paternal line can act as a form of control in studies of maternal effects.[9,10] However, this was not our reason for analysing potential paternal exposure transgenerational effects in our earlier paper[8] or in the present analysis of smoking mothers. They were instigated by studies from Sweden based on samples of individuals born in the town of Överkalix. Their longevity and other health outcomes were linked to detailed historical records of harvests experienced by their ancestors. Although most of the emphasis in the Överkalix study was concerned with exposures in mid-childhood[11,12] the studies of three cohorts pooled together have demonstrated effects of exposures of the paternal grandmother [PGM] prenatally to times of very poor harvests on significantly increased mortality of her granddaughters but not her grandsons.[13] Thus, the presumed effect is from the *in utero* exposure of the paternal grandmother to her son and subsequently to his daughter. Such transgenerational effects are now well supported by rodent experiments showing male-line transmissions and often demonstrating sex-specific

For peer review only

transmission on outcomes,[14-17] some focusing on imprinted gene expression in descendants[18] and others on associated epigenetic changes,[19-21] although no transgenerational signal itself has been clearly defined.[22]

Our earlier transgenerational study of intrauterine exposure of non-smoking mothers did not consider relationships with fetal growth if the study mother was also a smoker.[8] Here we use the same cohort, the Avon Longitudinal Study of Parents and Children [ALSPAC], to investigate the fetal growth of offspring of smoking mothers only – comparing the offspring of mothers and fathers who were themselves exposed to cigarette smoke *in utero* with those who were not exposed in this way. The only study that we are aware of that has looked at an aspect of this question compared the birth weight of the grandchildren comparing those born to mothers who smoked according to whether they themselves had been exposed to their own mothers' smoking *in utero*.[23] Altogether they reported a decrease of 70g if the grandmother had also smoked during pregnancy. The authors did not assess whether this difference was merely a function of variation in the amount smoked by the study mother. Nor did they assess whether there was any effect discernible with the prenatal smoke exposure of the study father, or whether there was any difference between the effects depending on the sex of the offspring.

The current study was therefore carried out to assess whether there is indeed a reduction in the growth of the fetus of a smoking mother if her own mother smoked, and/or whether exposure of the father in utero has any effect on the growth of the child of the smoking mother. In line with the evidence of the accumulating transgenerational human and animal data outlined above, we hypothesise that any effects will differ between boy and girl infants.

For peer review only

METHODS

Ethics statement

Ethical approval for the ALSPAC study was obtained from the ALSPAC Law and Ethics Committee and the three Avon-based Local Research Ethics Committees: Bristol and Weston Health Authority: E1808 Children of the Nineties: Avon Longitudinal Study of Pregnancy and Childhood (ALSPAC) (28th November 1989). Southmead Health Authority: 49/89 Children of the Nineties - "ALSPAC" (5th April 1990). Frenchay Health Authority: 90/8 Children of the Nineties. (28th June 1990). Written consent was obtained for all assays of biological samples. Ethics Committees considered voluntarily returned postal questionnaires as implied consent.

Study sample

The data used in these analyses were collected as part of the Avon Longitudinal Study of Parents and Children (ALSPAC), which was designed to assess the ways in which the environment interacts with the genotype to influence health and development.[24] Pregnant women, resident in the study area in south-west England with an expected date of delivery between 1st April 1991 and 31st December 1992, were invited to take part. About 80% of the eligible population did so.[25]

Information collected from the study parents during their study pregnancy included details of the maternal and paternal grandparents. Figure 1 illustrates the two pathways of possible influence of parental prenatal exposure to cigarette smoke on the study child that we investigate in this paper.

Figure 1. Diagram of intergenerational linkage, where MGM = maternal grandmother; PGM = paternal grandmother

For peer review only

The exposures

The women and their partners were sent a number of questionnaires during pregnancy.[26] These elicited information on their current smoking habits and those of their parents (i.e. the study grandparents). If they reported that their mothers had smoked, they were asked whether their mothers had smoked when expecting them – and, if so, were given the responses: yes / no / don't know from which to select. Thus the parents who replied 'don't know', had a mother who smoked but the parent was unsure whether she had smoked during her pregnancy. We have analysed these data in two ways: (a) assuming that all these women did smoke during pregnancy, and (b) omitting the 'don't knows' from the analyses and only analysing those definitely reported as smoking during the study pregnancy [this we have treated as a sensitivity analysis]. All mothers who themselves did not smoke during the study pregnancy were excluded from these analyses. Consequently we compared two groups of grandchildren: those whose grandmothers had smoked during the pregnancy resulting in their parent and whose mothers had also smoked during the pregnancy that resulted in the study child [MGM+M+ and PGM+M+] with those whose grandmothers had not smoked [MGM-M+ and PGM-M+] respectively. In these analyses all study mothers smoked during pregnancy. Analyses of fetal growth measures took account of the highest amount smoked by the mother during the study pregnancy, grouped as <10; 10-19; 20+ per day.

Possible confounders

Other data used in the analyses include the study mother's parity (as ascertained from the maternal report of previous pregnancies resulting in either a live- or still-birth, and coded as 0; 1+); gestation (completed weeks: 39+; 37-38; ≤36); mother's partner smoking at the start of pregnancy (primarily reported by the partner, but maternal report was used if the partner report was missing: yes; no); maternal age at the birth of the child (continuous);

For peer review only

housing tenure as a measure of socioeconomic background (owned or mortgaged; rented public housing; all other), maternal education (highest level of educational attainment – in five levels of increasing achievement), maternal alcohol consumption when the mother first felt the baby move (not at all; <1 glass per week; and one or more glasses per week).

Outcome measures

At delivery the baby was weighed to the nearest gram; ALSPAC study staff visited the two main delivery hospitals each day and measured the crown-heel length and head circumference of available infants in a standardised manner.[24] Body mass index (BMI) was calculated as birth weight/length^2 (g/m^2). In this study we have used BMI, rather than ponderal index (PI) as our measure of adiposity at birth; although it is traditional to use PI at birth, there is little literature to justify this. It has been suggested that the criteria used to choose whether to use PI or BMI should be a measure that is independent of length.[27] We have assessed which of the two measures is independent of length at each gestation among ALSPAC births and found that BMI satisfies the independence requirement more closely than PI.[8]

Statistical analyses

Multivariable linear regression models assessed the study children's adjusted mean birth weight, crown-heel length, head circumference and BMI by parental prenatal smoking exposure. All models were adjusted for parity, maternal education, amount mother smoked, paternal smoking in pregnancy and gestation with MGM-M+ (and PGM-M+) as the reference categories. Additional models adjusted for maternal age, housing tenure and maternal alcohol use as well as maternal birth weight.

For peer review only - http://bmjopen.bmj.com/site/about/guidelines.xhtml

Sensitivity analyses

In order to determine consistency of the findings, separate analyses were undertaken for primiparae and multiparae. In order to determine whether the head circumference results were biologically meaningful, we also used the fact that reduced head circumference is associated with lower levels of childhood IQ.[28] Childhood IQ was assessed by trained psychologists at age 8 years (56·2% of eligible children attended), with an abbreviated form of the Wechsler Intelligence Scale for Children (WISC-III).[29] This abbreviated form has been shown to be a valid method for use in research studies.[30]

RESULTS

In all, there were 3502 births to smoking mothers for whom data were available as to whether their own mothers had smoked when expecting them [Table 1]. Approximately half had such a history. Fewer women had information about the prenatal exposures of the father of their study child [n= 2354], but again exposure was approximately 50:50.

For peer review only

Table 1. The study sample of mothers who smoked during pregnancy

	MGM+	MGM-	PGM+	PGM-
**No. in study**	**1781**	**1721**	**1209**	**1145**
Maternal smoking in pregnancy (cigarettes per day)[a]				
<10	500 (28.0)	699 (40.6)	402 (33.3)	460 (40.2)
10-19	811 (45.6)	698 (40.6)	524 (43.3)	475 (41.5)
20+	469 (26.4)	324 (18.8)	283 (23.4)	209 (18.3)
	P < 0.001		P <0.001	
Parity[a]				
0	747 (43.3)	779 (46.1)	511 (43.3)	537 (47.8)
1+	980 (56.8)	910 (53.9)	668 (56.7)	586 (52.2)
	P = 0.092		P = 0.031	
Maternal education level[a]				
CSE or less	598 (39.6)	399 (26.3)	362 (33.8)	284 (27.6)
Vocational	195 (12.9)	184 (12.2)	144 (13.5)	126 (12.3)
O Level	493 (32.6)	548 (36.2)	367 (34.3)	357 (34.7)
A Level	181 (12.0)	291 (19.2)	156 (14.6)	195 (19.0)
Degree	45 (3.0)	93 (6.1)	41 (3.8)	66 (6.4)
	P<0.001		P<0.001	
Gestation(weeks)[a]				
39+	1328 (75.1)	1279 (74.6)	860 (71.5)	868 (75.9)
37/38	305 (17.3)	312 (18.2)	249 (20.7)	197 (17.2)
<37	135 (7.6)	123 (7.2)	94 (7.8)	78 (6.8)
	P = 0.070		P = 0.048	
Partner smoking[a]				
No	478 (29.7)	487 (30.3)	341 (28.3)	344 (30.0)
Yes	1130 (70.3)	1120 (69.7)	863 (71.7)	801 (70.0)
	P = 0.072		P = 0.360	
Housing tenure[a]				
Owned/mortgaged	736 (44.5)	965 (59.1)	583 (50.9)	677 (61.8)
nted public	628 (38.0)	406 (24.9)	386 (33.7)	247 (22.5)
Rented private/other	291 (17.6)	263 (16.1)	177 (15.5)	172 (15.7)
	P < 0.001		P < 0.001	
Maternal alcohol[a]				
Never	870 (50.9)	827 (49.6)	602 (51.7)	566 (51.5)
<1 glass per week	563 (32.9)	530 (31.8)	376 (32.3)	348 (31.6)
1+ glasses per week	277 (16.2)	311 (18.7)	186 (16.0)	186 (16.9)
	P = 0.170		P = 0.830	
Maternal age (yr)[b]	25.8 (5.1)	26.8 (5.1)	26.1 (5.1)	26.8 (5.0)
	P < 0.001		P = 0.003	
Maternal birthweight (kg)[b]	3.12 (0.67)	3.32 (0.63)	3.20 (0.65)	3.22 (0.67)
	P<0.001		P=0.570	

[a] n(%); [b] mean (SD); MGM maternal grandmother; PGM paternal grandmother; + smoked in pregnancy; - did not smoke in pregnancy

For peer review only

Comparison of data concerning the potential confounders [Table 1] indicates that if either grandmother had smoked prenatally, then the smoking study mother herself was more likely to be a heavy smoker, to have had lower educational attainment and to be younger; in addition the family was more likely to be living in rented public housing. Not surprisingly, the women who had been exposed in utero (i.e. MGM+) had considerably lower mean birth weight themselves (by 199g) than those not exposed (MGM-). There was no difference in prevalence of smoking by the study father if his own mother had smoked during pregnancy.

Table 2 compares the birth measurements of study children born to parents who had been exposed to smoking *in utero*. It can be seen that for the women who had themselves been exposed *in utero*, there was just one statistically significant unadjusted association in their progeny (a lower birth weight for girls), but that this was no longer significant upon adjustment. For paternal *in utero* exposure, however, there were several unadjusted associations [with girls' birth weight and birth length, and with boys' birth length and head circumference]. On adjustment, the association with head circumference remained with a 0.35cm reduction [95% CI -0.57, -0.14] for boys [P = 0.001], but the association for girls was quite different: +0.08 [95%CI -0.11, +0.28]; (P for interaction = 0.006).

For peer review only

Table 2. Mean difference (P value) [95% CI] in birth measurements of children born to smoking mothers, comparing those where the child's grandmother had smoked with those who had not

	MGM+ M+ v. MGM- M+		PGM+ M+ v. PGM- M+	
	Unadjusted	Adjusted[a]	Unadjusted	Adjusted[a]
**BIRTHWEIGHT (g)**				
Boy	-13 (0.65)	-29 (0.24)	-55 (0.11)	-50 (0.074)
	[-69, +43]	[-77, +19]	[-123, +13]	[-104, +5]
Girl	**-63 (0.022)**	-31 (0.22)	**-88 (0.010)**	-11 (0.28)
	**[-116, -9]**	[-81, +18]	**[-155, -22]**	[-67, +45]
**BIRTH LENGTH (cm x100)**				
Boy	+8 (0.59)	-0 (1.00)	**-37 (0.035)**	-29 (0.070)
	[-20, +36]	[-28, +28]	**[-72, -3]**	[-61, +3]
Girl	-11 (0.44)	+7 (0.59)	**-37 (0.037)**	+1 (0.96)
	[-39, +17]	[-20, +35]	**[-71, -2]**	[-31, +33]
**HEAD CIRCUMFERENCE (cm x100)**				
Boy	+4 (0.66)	-3 (0.74)	**-35 (0.003)**	**-35 (0.001)**
	[-14, +23]	[-22, +16]	**[-59, -12]**	**[-57, -14]**
Girl	-9 (0.28)	-3 (0.76)	-17 (0.107)	+8 (0.39)
	[-27, +8]	[-19, +14]	[-39, +4]	[-11, +28]
**BMI ((kg/m^2)*10)**				
Boy	-0.3 (0.73)	-0.8 (0.37)	-1.2 (0.24)	-1.0 (0.31)
	[-1.9, +1.3]	[-2.5, +0.9]	[-3.1, +0.8]	[-2.9, +0.9]
Girl	-1.8 (0.41)	-1.3 (0.15)	-2.1 (0.056)	-0.6 (0.57)
	[-3.5, -0.1]	[-3.1, +0.5]	[-4.2, +0.1]	[-2.6, +1.5]

[a]Adjusted for maternal parity, maternal education, partner smoked in pregnancy, gestation of study child and the amount the mother smoked

[N.B. the data for birth length and head circumference are given in cm x 100 so as to aid viewing]

For peer review only

Sensitivity analyses

The analyses were repeated for primiparae and multiparae separately [Supplementary Tables 1 and 2]. The only significant association that remained after adjustment concerned the head circumference of the study sons. The effect sizes were similar for each parity group: for primiparae the effect size was -0.34 [95%CI -0.66, -0.02]cm, P = 0.036; for multiparae the adjusted effect size was similar at -0.35 [95% CI -0.64, -0.06]cm, P= 0.017. Again there were significant interactions with the sex of the child.

Since this association with head circumference was consistent but unexpected, and since there is evidence that birth head circumference is associated with childhood IQ,[30] we used the same study methodology to assess whether a similar association was apparent between paternal prenatal exposure and childhood IQ. Table 3 demonstrates that there was indeed a reduction in adjusted mean IQ of 2.90 points [95% CI -5.72, -0.08] (P = 0.044) for sons of exposed fathers, but no such association for daughters, although the interaction with sex was not statistically significant. Full scale IQ is made up of the sum of two components [performance IQ and verbal IQ] that are, in general, known to have different genetic and environmental components.[31] We therefore have analysed the data to assess whether the associations with paternal grandmothers' smoking during pregnancy are associated with one of these components in particular. We found that paternal exposure *in utero* had a greater effect on his son's verbal IQ [mean adjusted difference -3.65 points; 95% CI -6.60, -0.70], but with little difference in performance IQ [mean -1.40 [95% CI -4.39, +1.60] points.

For peer review only

Table 3. Mean difference [95% CI] in birth measurements of children born to smoking mothers, comparing those where the child's grandmother had smoked with those who had not.

	MGM+M+ v. MGM-M+		PGM+M+ v. PGM-M+	
	Unadjusted	Adjusted[a]	Unadjusted	Adjusted[a]
**Total IQ**				
Boys	**-3.87**	-2.45	**-4.00**	**-2.90**
95% CI	**[-6.34, -1.40]**	[-4.96, +0.07]	**[-6.92, -1.08]**	**[-5.72, -0.08]**
P value	**0.002**	0.057	**0.007**	**0.044**
Number	694	612	507	482
Girls	**-2.50**	-0.40	**-3.03**	-1.36
95% CI	**[-4.90, -0.11]**	[-2.85, +2.05]	**[-5.78, -0.28]**	[-4.07, +1.36]
P value	**0.041**	0.749	**0.031**	0.327
Number	617	551	456	436
**Performance IQ**				
Boys	**-2.64**	-1.48	-2.44	-1.40
95% CI	**[-5.20, -0.08]**	[-4.20, +1.24]	[-5.40, +0.50]	[-4.39, +1.60]
P value	**0.043**	0.287	0.104	0.360
Number	698	616	510	485
Girls	-2.46	-0.50	**-3.03**	-1.74
95% CI	[-5.01, +0.09]	[-3.19, +2.19]	**[-5.88, -0.18]**	[-4.69, +1.20]
P value	0.059	0.716	**0.037**	0.245
N	619	552	457	436
**Verbal IQ**				
Boys	**-3.75**	-2.40	**-4.73**	**-3.65**
95% CI	**[-6.30, -1.21]**	[-5.00, +1.20]	**[-7.81, -1.66]**	**[-6.60, -0.70]**
P value	**0.004**	0.070	**0.003**	**0.015**
Number	697	615	509	484
Girls	-1.98	-0.15	-2.48	-0.81
95% CI	[-4.37, +0.42]	[-2.60, +2.30]	[-5.28, +0.32]	[-3.54, +1.92]
P value	0.106	0.906	0.082	0.561
Number	617	551	456	436

[a]Adjusted for maternal education, parity, partner smoked in pregnancy, gestation of study child and the amount the mother smoked.

For peer review only

DISCUSSION

We investigated whether the parents' exposure *in utero* to their own mothers' smoking was associated with differences in fetal growth among women who smoked in pregnancy, and showed an association between paternal *in utero* exposure and a reduced head circumference in his sons, but not in his daughters. This was an unexpected finding. A series of sensitivity analyses showed the effect to be almost identical in children born to primiparae and to those born to multiparae. We assessed whether there was confirmatory evidence of an impact on brain size by looking at the IQ of the children. We found a significant reduction in total IQ in 8-year-old boys (but not girls) whose paternal grandmother smoked during the pregnancy resulting in the study child's father. The IQ effect size was similar in both parity groups and was still present when birth head circumference was taken into account [data not shown]. We showed a stronger association with verbal IQ than performance IQ. To our knowledge there have been no previous studies that have considered any effects of paternal exposure to smoking *in utero* on his offspring.

Strengths and limitations

There are a number of limitations to this study: (i) Details of smoking of parents and grandparents depend on parental self-report – however there is considerable information to indicate that adults are unlikely to lie about smoking habits, especially when using anonymised self-completion questionnaires;[32] here we have shown that the mean birth weight of the study mothers who had reported that their own mothers had smoked when they were *in utero* was 199g lower than that of those who had reported that their mother did not smoke at that time, which was about the expected order of difference if the mothers had reported accurately; (ii) although the amount the parents smoked was reported, there was no

For peer review only

estimate requested for the amount smoked by the grandmothers when pregnant with the study parent – this may have been associated with the outcome, but it is difficult to postulate how such effects might differ between the sexes of the study children; (iii) although the ALSPAC study is large, the numbers of women who smoked throughout pregnancy and for whom details are available on the grandmothers' smoking are reduced and consequently the statistical power is relatively low. Among the strengths of this study are the following: (a) it tested a prior hypothesis that early life exposures can have phenotypic effects down the paternal line with sex-specific outcomes; (b) the information on grandparental and parental smoking was collected prior to the birth of the study child, and consequently cannot have been biased by knowledge of fetal size; (c) birth length and head circumference were ascertained by trained measurers using standard techniques, as opposed to the generally inaccurate methods used in most delivery units; (d) IQ was measured using standard methodology by trained psychologists; (e) the study was based on a relatively large population sample, and results are therefore likely to be generalisable.

Meaning of the study

Our previous study of non-smoking mothers, looking at the effect of parental exposure *in utero*, found their sons were larger at birth (both weight and BMI) if the maternal grandmother had smoked in the pregnancy that resulted in the study mother. There was no discernible effect of paternal prenatal exposure on the study child's birth weight or BMI; however there was a slight *increase* in head circumference among the boys born to fathers who had been exposed *in utero* [mean difference +0.08 cm; 95% CI -0.03, +0.19].[8]

Attributing the smaller head circumference in boys of smoking mothers to the prenatal exposure of the father through his own mother's smoking, raises the question of possible

For peer review only

mechanisms. How might the information be transmitted via his sperm or in some other way? As we noted in the introduction to this paper there is increasing evidence that exposures, especially in early life, can lead to enduring changes in the epigenome that, in turn, can modify gene expression. Whilst transgenerational epigenetic inheritance remains controversial, at least in humans,[33] the phenomenon of genomic imprinting establishes the principle that epigenetic marks such as DNA methylation placed in one generation can influence gene expression in the next. One such imprinted gene is the Insulin Growth Factor 2 (*IGF2*) which is expressed only from the paternally-derived chromosome 11, the maternal copy being epigenetically silenced. *IGF2* encodes an endocrine and autocrine/paracrine acting factor important in directing growth during prenatal development.[34,35] Maternal smoking has been shown to be associated with a 5% higher DNA methylation level at the *IGF2* DMR (differentially methylated region) in the newborn infant,[5] and interestingly in the context of our study, this methylation shift is specific to male offspring. Thus it is possible that the study father's *IGF2* DMR had been epigenetically modified (including in his fetal testes) by his mother's smoking throughout pregnancy. Furthermore it is plausible that this epigenetic state could be transmitted via his sperm to the study offspring. Imprinted gene regions tend to escape the usual widespread erasure of DNA methylation from the paternally-derived genome in the pre-implantation embryo soon after fertilisation.[36] In support of paternal effects generally, there is a report of hypomethylation at the *IGF2* DMR in umbilical cord blood being associated with paternal obesity suggesting a preconceptional impact of the obesity (and/or exposures related to it) on the reprogramming of imprint marks during spermatogenesis.[37]

For peer review only

Unanswered questions and further research

There are few studies on head circumference at birth. This is possibly because the measurements are generally inaccurate. Pilot studies before the start of the ALSPAC study had demonstrated that it was usually the student midwife who was given the task of measuring the circumference of the baby's head; she tended to have had little or no training and the measurements made were grossly inaccurate. For this study we only used measurements that were made by our own staff, after detailed training and with repeated validation over time. Detailed studies with accurate data to test the hypothesis raised by the results of this study are needed.

In conclusion

When the mother is a smoker, we found no effect of her own tobacco exposure *in utero* on the fetal growth of her children. However, when the mother is a smoker, paternal exposure *in utero* is associated with a reduced head circumference at birth and IQ at 8 years in sons, but not daughters. We had no prior hypothesis that head circumference would be associated, particularly among sons, so these results must be considered as hypothesis generating, and require testing in further longitudinal data sets.

For peer review only

Acknowledgements

We are extremely grateful to all the families who took part in this study, the midwives for their help in recruiting them, and the whole ALSPAC team, which includes interviewers, computer and laboratory technicians, clerical workers, research scientists, volunteers, managers, receptionists and nurses.

Authors' contributions: MP and JG had the idea; KN, SG and LLM carried out the statistical analyses; JG and MP wrote the first draft and all authors contributed to the final manuscript.

Data sharing: The ALSPAC data can be used by bona fide researchers. See the study website for the conditions: http://www.bristol.ac.uk/alspac/researchers/

Funding: The UK Medical Research Council (www.mrc.ac.uk), the Wellcome Trust (www.Wellcome.ac.uk) and the University of Bristol (www.bristol.ac.uk) currently provide core support for ALSPAC. The statistical analyses for this project were undertaken with funding from the Medical Research Council [grant no. G1100226]. The funders had no role in study design, data collection, statistical analysis, decision to publish, or preparation of the manuscript.

Competing interests: The authors have no competing interests.

For peer review only

References

1.Barker DJ. In utero programming of chronic disease. *Clin Sci* 1998;**95**:115-128.

2.Gluckman PD, Hanson MA, Buklijas T, et al. Epigenetic mechanisms that underpin metabolic and cardiovascular disease. *Nat Rev Endocrinol* 2009;**5**:401- 408. doi:10.1038/nrendo.2009.102

3.Relton C, Davey-Smith G, Ozanne S. Developmental Epigenetic Programming in Diabetes and Obesity. In: Jirtle R, Tyson E, eds. Environmental Epigenomics in Health and Disease; Epigenetics and Complex Diseases. Berlin Heidelberg: Springer 2013:235-253.

4.Breton CV, Byun HM, Wenten M, et al. Prenatal tobacco smoke exposure affects global and gene-specific DNA methylation. *Am J Respir Crit Care Med* 2009;**180**:462-467. Doi. 10.1164/rccm.200901-0135OC

5.Murphy SK, Adigun A, Huang Z, et al. Gender-specific methylation differences in relation to prenatal exposure to cigarette smoke. *Gene* 2012;**15**:36-43. doi.org/10.1016/j.gene.2011.11.062.

6.Joubert BR, Håberg SE, Nilsen RM, et al. 450K epigenome-wide scan identifies differential DNA methylation in newborns related to maternal smoking during pregnancy. *Environ Health Perspect* 2012;**120**:1425-1431. 10.1289/ehp.1205412.

7. Lee KKW, Pausova Z. Cigarette smoking and DNA methylation. *Front Genet* 2013;**4**:132. doi: 10.3389/fgene.2013.00132

8.Miller LL, Pembrey M, Davey Smith G, et al. Is the growth of the fetus of a non-smoking mother influenced by the smoking of either grandmother while pregnant? *PLoS ONE* 2014; In press.

9.Davey Smith G. Assessing intrauterine influences on offspring outcomes: Can epidemiological studies yield robust outcomes? *Basic Clin Pharmacol Toxicol* 2008;**102**:245-256. DOI: 10.1111/j.1742-7843.2007.00191.x

10.Macdonald-Wallis C, Tobias JH, Davey Smith G, et al. Parental smoking during pregnancy and offspring bone mass at age 10 years: findings from a prospective birth cohort. *Osteoporos Int* 2011; **22**:1809-1819. Doi: 10.1007/s00198-010-1415-y

11.Bygren LO, Kaati G, Edvinsson S. Longevity determined by ancestors' over nutrition during their slow growth period. *Acta Biotheor* 2001;**49**:53-59. Doi. 10.1023/A:1010241825519

12.Kaati G, Bygren LO, Edvinsson S. Cardiovascular and diabetes mortality determined by nutrition during parents' and grandparents' slow growth period. *Eur J Hum Genet* 2002;**10**:682-688. DOI:10.1038/sj.ejhg.5200859

13.Pembrey ME, Bygren LO, Kaati G et al. Sex-specific, male-line transgenerational responses in humans. *Eur J Hum Genet* 2006;**14**:159–166. doi:10.1038/sj.ejhg.5201538

14.Anway MD, Cupp AS, Uzumcu M, et al. Epigenetic transgenerational actions of endocrine disruptors and male fertility. *Science* 2005;**308**:1466-1469. DOI: 10.1126/science.1108190

15.Franklin TB, Russig H, Weiss IC et al. Epigenetic transmission of the impact of early stress across generations. *Biol Psychiatr* 2010;**68**:408-415.doi10.1016/j.biopsych.2010.05.036

16.Ng SF, Lin RC, Laybutt DR et al. Chronic high-fat diet in fathers programs β-cell dysfunction in female rat offspring. *Nature* 2010;**467**:963-966. doi:10.1038/nature09491

17.Zeybel M, Hardy T, Wong YK, et al. Multigenerational epigenetic adaptation of the hepatic wound-healing response. *Nat Med* 2012;**18**:1369–1377. doi:10.1038/nm.2893

For peer review only

18.Dunn GA, Bale TL. Maternal high-fat diet effects on third-generation female body size via the paternal lineage. *Endocrinol* 2011;**152**:2228-2236. Doi:10.1210/en.2010.1461

19.Burdge GC, Slater-Jefferies J, Torrens C, et al. Dietary protein restriction of pregnant rats in the F0 generation induces altered methylation of hepatic gene promoters in the adult male offspring in the F1 and F2 generations. *Br J Nutr* 2007;**97**:435-439. Doi 10.1017/S0007114507352392

20.Carone BR, Fauquier L, Habib N, et al. Paternally induced transgenerational environmental reprogramming of metabolic gene expression in mammals. *Cell* 2010;**143**:1084-1096. Doi:10.1016/j.cell.2010.12.008

21.Dias BG, Ressler KJ. Parental olfactory experience influences behavior and neural structure in subsequent generations. *Nat Neurosci* 2014;**17**:89-96. Doi: doi:10.1038/nn.3594

22.Daxinger L, Whitelaw E. Understanding transgenerational epigenetic inheritance via the gametes in mammals. *Nat Rev Genet* 2012;**31**:153-162. doi:10.1038/nrg3188

23.Misra DP, Astone N, Lynch CD. Maternal smoking and birth weight: interaction with parity and mother's own in utero exposure to smoking. *Epidemiol* 2005;**16**:288–293. doi: 10.1097/01.ede.0000158198.59544.cf

24.Golding J, Pembrey M, Jones R. ALSPAC–the Avon Longitudinal Study of Parents and Children. I. Study methodology. *Paediatr Perinatal Epidemiol* 2001;**15**:74–87. DOI: 10.1046/j.1365-3016.2001.00325.x

25.Boyd A, Golding J, Macleod J, et al. Cohort Profile: The 'Children of the 90s'–the index offspring of the Avon Longitudinal Study of Parents and Children. *Int J Epidemiol* 2012;**42**:111-127. doi. 10.1093/ije/dys064

26. ALSPAC web: http://www.bristol.ac.uk/alspac/researchers/resources-available/data-details/questionnaires/. Accessed 2 December 2013.

For peer review only

27.Cole TJ, Henson GL, Tremble JM, et al. Birthweight for length: ponderal index, body mass index or Benn index? *Ann Hum Biol* 1997;**24**:289–298. doi:10.1080/03014469700005032

28.Gale CR, O'Callaghan FJ, Bredow M, et al. The influence of head growth in fetal life, infancy, and childhood on intelligence at the ages of 4 and 8 years. *Pediatr* 2006;**118**:1486-1492. doi: 10.1542/peds.2005-2629

29.Wechsler D, Golombok S, Rust J. WISC-IIIUK Wechsler Intelligence Scale for Children. Sidcup, UK: Psychological Corp 1992.

30. Gunnell D, Miller LL, Rogers I, et al. Association of Insulin-like Growth Factor I and Insulin-like Growth Factor–Binding Protein-3 With Intelligence Quotient Among 8-to 9-Year-Old Children in the Avon Longitudinal Study of Parents and Children. *Pediatr* 2005; **116**:e681-e686. doi: 10.1542/peds.2004-2390

31. Dickens WT, Flynn JR. Heritability estimates versus large environmental effects: the IQ paradox resolved. *Psychol Rev* 2001;**108**:346. Doi:10.1037/0033-295X.108.2.346

32. Klebanoff MA, Levine RJ, Clemens JD, et al. Serum cotinine concentration and self-reported smoking during pregnancy. *Am J Epidemiol* 1998;**148**:259-262.

33.Grossniklaus U, Kelly B, Ferguson-Smith AC, et al. Transgenerational epigenetic inheritance: how important is it? *Nat Rev Genet* 2013;**14**:228-235. doi:10.1038/nrg3435

34.LeRoith D, Lowe WL. Growth factors. In: Melmed S, Conn PM, eds. Endocrinology: Basic and Clinical Principles, 2nd edition. Totowa, New Jersey: Humana Press 2005:85-91.

35 Demetriou C, Abu-Amero S, Thomas AC, et al. Paternally expressed, imprinted Insulin-1 like growth factor-2 in chorionic villi correlates significantly with birth weight. *PLoS ONE* 2014;doi:10.1371/journal.pone.0085454.

For peer review only

36.Seisenberger S, Peat JR, Hore TA, et al. Reprogramming DNA methylation in the mammalian life cycle: building and breaking epigenetic barriers. *Philos Trans R Soc Lond B Biol Sci* 2013;**368**: 20110330. doi: 10.1098/rstb.2011.0330.

37.Soubry A, Schildkraut JM, Murtha A, et al. Paternal obesity is associated with IGF2 hypomethylation in newborns: results from a Newborn Epigenetics Study (NEST) cohort. *BMC Med* 2013;29:doi:10.1186/1741-7015-11-29

For peer review only

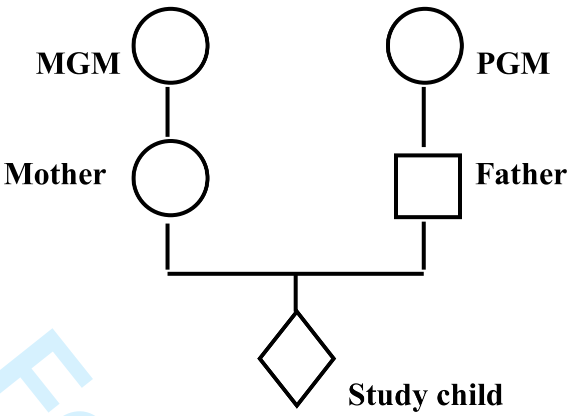

For peer review only

Supplementary Table 1. Mean difference (P value) [95% CI] in birth measurements of children born to smoking mothers, comparing those where the child's grandmother had smoked with those who had not when the child is the mother's firstborn

	MGM+ M+ v. MGM- M+		PGM+ M+ v. PGM- M+	
	Unadjusted	Adjusted[a]	Unadjusted	Adjusted[a]
**BIRTHWEIGHT (g)**				
Boy	-21 (0.64)	+5 (0.90)	-66 (0.22)	-51 (0.22)
	[-109, +67]	[-71, +80]	[-172, +40]	[-134, +31]
Girl	-79 (0.057)	-15 (0.69)	-86 (0.077)	-17 (0.69)
	[-160, +2]	[-92, +61]	[-181, +9]	[-101, +67]
**BIRTH LENGTH (cm x100)**				
Boy	+12 (0.58)	+22 (0.27)	-31 (0.22)	-33 (0.12)
	[-29, +52]	[-17, +61]	[-82, +19]	[-75, +09]
Girl	-26 (0.21)	+12 (0.58)	-34 (0.17)	-9 (0.71)
	[-66, +14]	[-31, +54]	[-84, +15]	[-57, +39]
**HEAD CIRCUMFERENCE (cm x100)**				
Boy	+6 (0.69)	+7 (0.62)	-37 (0.051)	**-34 (0.036)**
	[-23, +34]	[-21, +35]	[-74, +00]	**[-66, -2]**
Girl	-23 (0.068)	+1 (0.94)	-12 (0.46)	+6 (0.69)
	[-48, +2]	[-25, +27]	[-43, +20]	[-23, +35]
**BMI ((kg/m^2)x10)**				
Boy	-0.6 (0.59)	-0.1 (0.92)	-0.6 (0.65)	-1.1 (0.37)
	[-2.8, +1.6]	[-2.3, +2.1]	[-3.2, +2.0]	[-3.5, +1.3]
Girl	-2.6 (0.061)	-0.7 (0.64)	-2.6 (0.13)	-1.6 (0.35)
	[-5.2, +0.1]	[-3.6, +2.2]	[-6.0, +0.8]	[-5.0, +1.8]

[a]Adjusted for maternal education, partner smoked in pregnancy, gestation of study child and the amount the mother smoked

[N.B. the data for birth length and head circumference are given in cm x 100 so as to aid viewing]

For peer review only

Supplementary Table 2. Mean difference $^{(P\ value)}$ [95% CI] in birth measurements of children born to smoking mothers, comparing those where the child's grandmother had smoked with those who had not when the child is NOT the mother's firstborn

	MGM+ M+ v. MGM- M+		PGM+ M+ v. PGM- M+	
	Unadjusted	Adjusted[a]	Unadjusted	Adjusted[a]
**BIRTHWEIGHT (g)**				
Boy	-16 (0.67)	-52 (0.099)	-84 (0.061)	-51 (0.17)
	[-88, +57]	[-115, +10]	[-172, +4]	[-125, +22]
Girl	-54 (0.15)	+41 (0.22)	**-106 (0.027)**	-1 (0.98)
	[-127, +19]	[-108, +25]	**[-199, -12]**	[-77, +75]
**BIRTH LENGTH (cm x100)**				
Boy	+1 (0.96)	-15 (0.44)	**-52 (0.033)**	-26 (0.27)
	[-38, +40]	[-5, +24]	**[-101, -4]**	[-73, +21]
Girl	+1 (0.97)	+4 (0.82)	-5 (0.074)	+14 (0.53)
	[-39, +40]	[-32, +41]	[-95, +4]	[-30, +57]
**HEAD CIRCUMFERENCE (cm x100)**				
Boy	+4 (0.74)	-9 (0.47)	**-38 (0.016)**	**-35 (0.017)**
	[-21, +30]	[-35, +16]	**[-69, -7]**	**[-64, -6]**
Girl	-0 (0.98)	-2 (0.86)	-23 (0.12)	+11 (0.39)
	[-24, +23]	[-23, +19]	[-53, +6]	[-15, +37]
**BMI ((kg/m^2)x10)**				
Boy	-0.9 (0.45)	-1.2 (0.34)	-2.3 (0.11)	-1.1 (0.46)
	[-3.2, +1.4]	[-0.37, +1.3]	[-5.2, +0.5]	[-4.0, +1.8]
Girl	-1.1 (0.32)	-1.4 (0.21)	-2.1 (0.12)	+0.5 (0.87)
	[-3.4, +1.1]	[-3.7, +0.8]	[-4.8, +0.5]	[-2.0, +3.0]

[a]Adjusted for maternal education, partner smoked in pregnancy, gestation of study child and the amount the mother smoked

[N.B. the data for birth length and head circumference are given in cm x 100 so as to aid viewing]

For peer review only

STROBE Statement—checklist of items that should be included in reports of observational studies

	Item No	Recommendation
**Title and abstract**	1	(*a*) Indicate the study's design with a commonly used term in the title or the abstract
		(*b*) Provide in the abstract an informative and balanced summary of what was done and what was found
**Introduction**		
Background/rationale	2	Explain the scientific background and rationale for the investigation being reported
Objectives	3	State specific objectives, including any prespecified hypotheses
**Methods**		
Study design	4	Present key elements of study design early in the paper
Setting	5	Describe the setting, locations, and relevant dates, including periods of recruitment, exposure, follow-up, and data collection
Participants	6	(*a*) *Cohort study*—Give the eligibility criteria, and the sources and methods of selection of participants. Describe methods of follow-up<br>*Case-control study*—Give the eligibility criteria, and the sources and methods of case ascertainment and control selection. Give the rationale for the choice of cases and controls<br>*Cross-sectional study*—Give the eligibility criteria, and the sources and methods of selection of participants
		(*b*) *Cohort study*—For matched studies, give matching criteria and number of exposed and unexposed<br>*Case-control study*—For matched studies, give matching criteria and the number of controls per case
Variables	7	Clearly define all outcomes, exposures, predictors, potential confounders, and effect modifiers. Give diagnostic criteria, if applicable
Data sources/ measurement	8*	For each variable of interest, give sources of data and details of methods of assessment (measurement). Describe comparability of assessment methods if there is more than one group
Bias	9	Describe any efforts to address potential sources of bias
Study size	10	Explain how the study size was arrived at
Quantitative variables	11	Explain how quantitative variables were handled in the analyses. If applicable, describe which groupings were chosen and why
Statistical methods	12	(*a*) Describe all statistical methods, including those used to control for confounding
		(*b*) Describe any methods used to examine subgroups and interactions
		(*c*) Explain how missing data were addressed
		(*d*) *Cohort study*—If applicable, explain how loss to follow-up was addressed<br>*Case-control study*—If applicable, explain how matching of cases and controls was addressed<br>*Cross-sectional study*—If applicable, describe analytical methods taking account of sampling strategy
		(*e*) Describe any sensitivity analyses

Continued on next page

For peer review only

**Results**		
Participants	13*	(a) Report numbers of individuals at each stage of study—eg numbers potentially eligible, examined for eligibility, confirmed eligible, included in the study, completing follow-up, and analysed
		(b) Give reasons for non-participation at each stage
		(c) Consider use of a flow diagram
Descriptive data	14*	(a) Give characteristics of study participants (eg demographic, clinical, social) and information on exposures and potential confounders
		(b) Indicate number of participants with missing data for each variable of interest
		(c) *Cohort study*—Summarise follow-up time (eg, average and total amount)
Outcome data	15*	*Cohort study*—Report numbers of outcome events or summary measures over time
		Case-control study—Report numbers in each exposure category, or summary measures of exposure
		Cross-sectional study—Report numbers of outcome events or summary measures
Main results	16	(*a*) Give unadjusted estimates and, if applicable, confounder-adjusted estimates and their precision (eg, 95% confidence interval). Make clear which confounders were adjusted for and why they were included
		(*b*) Report category boundaries when continuous variables were categorized
		(*c*) If relevant, consider translating estimates of relative risk into absolute risk for a meaningful time period
Other analyses	17	Report other analyses done—eg analyses of subgroups and interactions, and sensitivity analyses
**Discussion**		
Key results	18	Summarise key results with reference to study objectives
Limitations	19	Discuss limitations of the study, taking into account sources of potential bias or imprecision. Discuss both direction and magnitude of any potential bias
Interpretation	20	Give a cautious overall interpretation of results considering objectives, limitations, multiplicity of analyses, results from similar studies, and other relevant evidence
Generalisability	21	Discuss the generalisability (external validity) of the study results
**Other information**		
Funding	22	Give the source of funding and the role of the funders for the present study and, if applicable, for the original study on which the present article is based

*Give information separately for cases and controls in case-control studies and, if applicable, for exposed and unexposed groups in cohort and cross-sectional studies.

Note: An Explanation and Elaboration article discusses each checklist item and gives methodological background and published examples of transparent reporting. The STROBE checklist is best used in conjunction with this article (freely available on the Web sites of PLoS Medicine at http://www.plosmedicine.org/, Annals of Internal Medicine at http://www.annals.org/, and Epidemiology at http://www.epidem.com/). Information on the STROBE Initiative is available at www.strobe-statement.org.

For peer review only

Is the growth of the child of a smoking mother influenced by the father's prenatal exposure to tobacco? A hypothesis generating longitudinal study

Journal:	*BMJ Open*
Manuscript ID:	bmjopen-2014-005030.R1
Article Type:	Research
Date Submitted by the Author:	18-Jun-2014
Complete List of Authors:	Pembrey, Marcus; University of Bristol, Social and Community Medicine<br>Northstone, Kate; University of Bristol, Centre for Child and Adolescent Health<br>Gregory, Steven; University of Bristol, Social and Community Medicine<br>Miller, Laura; University of Bristol, Social and Community Medicine<br>Golding, Jean; University of Bristol, Centre for Child and Adolescent Health
**Primary Subject Heading**:	Epidemiology
Secondary Subject Heading:	Public health
Keywords:	Transgenerational effects, ALSPAC, grandmothers' prenatal smoking, birth head circumference

For peer review only

Is the growth of the child of a smoking mother influenced by the father's prenatal exposure to tobacco? A hypothesis generating longitudinal study

Marcus Pembrey[abc], Kate Northstone[a], Steven Gregory[ab], Laura L Miller[a], Jean Golding[ab]

[a]School of Social and Community Medicine, [b]Centre for Child and Adolescent Health, University of Bristol, Bristol and [c] Institute of Child Health, University College London, London, UK

For peer review only

Key Words: ALSPAC, transgenerational effects, grandmothers' prenatal smoking, birth head circumference, IQ

Correspondence to:

Professor Jean Golding,
Centre for Child and Adolescent Health,
School of Social and Community Medicine,
Barley House,
Oakfield Grove
Bristol BS8 2BN,
UK

Email: jean.golding@bristol.ac.uk
Telephone: +44 (0)117 3310198 Fax: +44 (0)117 3313303

14.5.14 **Word Count: 3432**

Abstract

Objectives: Transgenerational effects of different environmental exposures is becoming of major interest, with rodent experiments focusing on epigenetic mechanisms. Previously we have shown that if the study mother is a non-smoker, there is increased mean birth weight, length and body mass index [BMI] in her sons if she herself had been exposed prenatally to her mother's smoking. The aim of this study was to determine whether the prenatal smoke exposure of either parent influenced the growth of the fetus of a smoking woman, and whether any effects were dependent on the fetal sex.

Design: Population based pre-birth cohort study.

Setting: Avon Longitudinal Study of Parents and Children.

Participants: Participants were residents of a geographic area with expected date of delivery between April 1991 and December 1992. Among pregnancies of mothers who smoked during pregnancy, data were available concerning maternal and paternal prenatal exposures to their own mother smoking for 3502 and 2354 respectively

Primary and secondary outcome measures:, Birth weight, length, BMI and head circumference.

Results: After controlling for confounders, there were no associations with birth weight, length or BMI. There was a strong adjusted association of birth head circumference among boys whose fathers had been exposed prenatally [mean difference -0.35cm; 95%CI -0.57,-0.14; P=0.001]. There was no such association with girls (interaction: P=0.006). Similar associations were found when primiparae and multiparae were analysed separately. In order to determine whether this was reflected in child development, we examined the relationships with IQ; we found that the boys born to exposed fathers had lower IQ scores on average, and that this was particularly due to the verbal component [mean difference in verbal IQ: -3.65; 95%CI -6.60,-0.70 points].

For peer review only

Conclusions: Head size differences concerning paternal fetal exposure to smoking were unexpected and, as such, should be regarded as hypothesis generating.

For peer review only

Strengths and limitations of this study

- This study is the first to examine the sex-specific fetal effects of parental prenatal exposure to cigarette smoking when the mother herself smoked during pregnancy.
- Data were collected on a population sample who completed questionnaires blind to the study hypotheses.
- Birth measurements were undertaken using trained staff with repeated validation.
- A variety of sensitivity analyses were undertaken, including separate analyses of primiparae and multiparae, as well as of follow-up of the offspring to determine whether the decrement in birth head circumference was reflected in childhood measurement of IQ. All were in accord with the initial finding.
- The limitation of the study is the failure to obtain comparable data to confirm or negate the study findings.

For peer review only

INTRODUCTION

Fetal programming via the mother's nutrition and other aspects of her environment is well-recognised as a contributor to adult morbidity and mortality[1] and some of these enduring effects are likely to be mediated by epigenetic mechanisms.[2,3] Studies have shown specific DNA methylation patterns in children whose mothers had smoked during pregnancy.[4-7] However, there have been few pre-conceptional transgenerational studies relating the fetal environment of either parent to the birth outcomes of their own children.

In an earlier study of non-smoking mothers, we found an increase in the birth weight and birth body mass index [BMI] of her sons if she had been exposed *in utero* to her own mothers' smoking, but there was no such effect if the study father had been exposed *in utero*.[8] This lack of paternal influence from his own intrauterine exposure was not unexpected. Indeed, it has been proposed that the paternal line can act as a form of control in studies of maternal effects.[9,10] However, this was not our reason for analysing potential paternal exposure transgenerational effects in our earlier paper[8] or in the present analysis of smoking mothers. They were instigated by studies from Sweden based on samples of individuals born in the town of Överkalix. Their longevity and other health outcomes were linked to detailed historical records of harvests experienced by their ancestors. Although most of the emphasis in the Överkalix study was concerned with exposures in mid-childhood[11,12] the studies of three cohorts pooled together have demonstrated effects of exposures of the paternal grandmother [PGM] prenatally to times of very poor harvests on significantly increased mortality of her granddaughters but not her grandsons.[13] Thus, the presumed effect is from the *in utero* exposure of the paternal grandmother to her son and subsequently to his daughter. Such transgenerational effects are now well supported by rodent experiments showing male-line transmissions and often demonstrating sex-specific

For peer review only

transmission on outcomes,[14-17] some focusing on imprinted gene expression in descendants[18] and others on associated epigenetic changes,[19-21] although no transgenerational signal itself has been clearly defined.[22]

Our earlier transgenerational study of intrauterine exposure of non-smoking mothers did not consider relationships with fetal growth if the study mother was also a smoker.[8] Here we use the same cohort, the Avon Longitudinal Study of Parents and Children [ALSPAC], to investigate the fetal growth of offspring of smoking mothers only – comparing the offspring of mothers and fathers who were themselves exposed to cigarette smoke *in utero* with those who were not exposed in this way. The only study that we are aware of that has looked at an aspect of this question compared the birth weight of the grandchildren comparing those born to mothers who smoked according to whether they themselves had been exposed to their own mothers' smoking *in utero*.[23] Altogether they reported a decrease of 70g if the grandmother had also smoked during pregnancy. The authors did not assess whether this difference was merely a function of variation in the amount smoked by the study mother. Nor did they assess whether there was any effect discernible with the prenatal smoke exposure of the study father, or whether there was any difference between the effects depending on the sex of the offspring.

The current study was therefore carried out to assess whether there is indeed a reduction in the growth of the fetus of a smoking mother if her own mother smoked, and/or whether exposure of the father in utero has any effect on the growth of the child of the smoking mother. In line with the evidence of the accumulating transgenerational human and animal data outlined above, we hypothesise that any effects will differ between boy and girl infants.

For peer review only

METHODS

Study sample

The data used in these analyses were collected as part of the Avon Longitudinal Study of Parents and Children (ALSPAC), which was designed to assess the ways in which the environment interacts with the genotype to influence health and development.[24] Pregnant women, resident in the study area in south-west England with an expected date of delivery between 1st April 1991 and 31st December 1992, were invited to take part. About 80% of the eligible population did so.[25]

Information collected from the study parents during their study pregnancy included details of the maternal and paternal grandparents. Figure 1 illustrates the two pathways of possible influence of parental prenatal exposure to cigarette smoke on the study child that we investigate in this paper.

Figure 1. Diagram of intergenerational linkage, where MGM = maternal grandmother; PGM = paternal grandmother

The exposures

The women and their partners were sent a number of questionnaires during pregnancy.[26] These elicited information on their current smoking habits and those of their parents (i.e. the study grandparents). If they reported that their mothers had smoked, they were asked whether their mothers had smoked when expecting them – and, if so, were given the responses: yes / no / don't know from which to select. Thus the parents who replied 'don't know', had a mother who smoked but the parent was unsure whether she had smoked during her pregnancy. We have analysed these data in two ways: (a) assuming that all these women did smoke during pregnancy, and (b) omitting the 'don't knows' from the analyses and only analysing those definitely reported as smoking during the study pregnancy [this we have

For peer review only

treated as a sensitivity analysis]. All mothers who themselves did not smoke during the study pregnancy were excluded from these analyses. Consequently we compared two groups of grandchildren: those whose grandmothers had smoked during the pregnancy resulting in their parent and whose mothers had also smoked during the pregnancy that resulted in the study child [MGM+M+ and PGM+M+] with those whose grandmothers had not smoked [MGM-M+ and PGM-M+] respectively. In these analyses all study mothers smoked during pregnancy. Analyses of fetal growth measures took account of the highest amount smoked by the mother during the study pregnancy, grouped as <10; 10-19; 20+ per day.

Possible confounders

Other data used in the analyses include the study mother's parity (as ascertained from the maternal report of previous pregnancies resulting in either a live- or still-birth, and coded as 0; 1+); gestation (completed weeks: 39+; 37-38; ≤36); mother's partner smoking at the start of pregnancy (primarily reported by the partner, but maternal report was used if the partner report was missing: yes; no); maternal age at the birth of the child (continuous); housing tenure as a measure of socioeconomic background (owned or mortgaged; rented public housing; all other), maternal education (highest level of educational attainment – in five levels of increasing achievement), maternal alcohol consumption when the mother first felt the baby move (not at all; <1 glass per week; and one or more glasses per week).

Outcome measures

At delivery the baby was weighed to the nearest gram; ALSPAC study staff visited the two main delivery hospitals each day and measured the crown-heel length and head circumference of available infants in a standardised manner.[24] Body mass index (BMI) was calculated as birth weight/length^2 (g/m^2). In this study we have used BMI, rather than

For peer review only

ponderal index (PI) as our measure of adiposity at birth; although it is traditional to use PI at birth, there is little literature to justify this. It has been suggested that the criteria used to choose whether to use PI or BMI should be a measure that is independent of length.[27] We have assessed which of the two measures is independent of length at each gestation among ALSPAC births and found that BMI satisfies the independence requirement more closely than PI.[8]

Pilot studies before the start of the ALSPAC study had demonstrated that it was usually the student midwife who was given the task of measuring the circumference of the baby's head; she tended to have had little or no training and the measurements made were grossly inaccurate. For this study we only used measurements that were made by our own staff, after detailed training and with repeated validation over time..

Statistical analyses

Multivariable linear regression models assessed the study children's adjusted mean birth weight, crown-heel length, head circumference and BMI by parental prenatal smoking exposure. All models were adjusted for parity, maternal education, amount mother smoked, paternal smoking in pregnancy and gestation with MGM-M+ (and PGM-M+) as the reference categories. Additional models adjusted for maternal age, housing tenure and maternal alcohol use as well as maternal birth weight.

Sensitivity analyses

In order to determine consistency of the findings, separate analyses were undertaken for primiparae and multiparae. In order to determine whether the head circumference results were biologically meaningful, we also used the fact that reduced head circumference is associated with lower levels of childhood IQ.[28] Childhood IQ was assessed by trained

For peer review only

psychologists at age 8 years (56·2% of eligible children attended), with an abbreviated form of the Wechsler Intelligence Scale for Children (WISC-III).[29] This abbreviated form has been shown to be a valid method for use in research studies.[30]

RESULTS

In all, there were 3502 births to smoking mothers for whom data were available as to whether their own mothers had smoked when expecting them [Table 1]. Approximately half had such a history. Fewer women had information about the prenatal exposures of the father of their study child [n= 2354], but again exposure was approximately 50:50.

For peer review only

Table 1. The study sample of mothers who smoked during pregnancy

	MGM+	MGM-	PGM+	PGM-
**No. in study**	**1781**	**1721**	**1209**	**1145**
Maternal smoking in pregnancy (cigarettes per day)[a]				
<10	500 (28.0)	699 (40.6)	402 (33.3)	460 (40.2)
10-19	811 (45.6)	698 (40.6)	524 (43.3)	475 (41.5)
20+	469 (26.4)	324 (18.8)	283 (23.4)	209 (18.3)
	P < 0.001		P <0.001	
Parity[a]				
0	747 (43.3)	779 (46.1)	511 (43.3)	537 (47.8)
1+	980 (56.8)	910 (53.9)	668 (56.7)	586 (52.2)
	P = 0.092		P = 0.031	
Maternal education level[a]				
CSE or less	598 (39.6)	399 (26.3)	362 (33.8)	284 (27.6)
Vocational	195 (12.9)	184 (12.2)	144 (13.5)	126 (12.3)
O Level	493 (32.6)	548 (36.2)	367 (34.3)	357 (34.7)
A Level	181 (12.0)	291 (19.2)	156 (14.6)	195 (19.0)
Degree	45 (3.0)	93 (6.1)	41 (3.8)	66 (6.4)
	P<0.001		P<0.001	
Gestation(weeks)[a]				
39+	1328 (75.1)	1279 (74.6)	860 (71.5)	868 (75.9)
37/38	305 (17.3)	312 (18.2)	249 (20.7)	197 (17.2)
<37	135 (7.6)	123 (7.2)	94 (7.8)	78 (6.8)
	P = 0.070		P = 0.048	
Partner smoking[a]				
No	478 (29.7)	487 (30.3)	341 (28.3)	344 (30.0)
Yes	1130 (70.3)	1120 (69.7)	863 (71.7)	801 (70.0)
	P = 0.072		P = 0.360	
Housing tenure[a]				
Owned/mortgaged	736 (44.5)	965 (59.1)	583 (50.9)	677 (61.8)
nted public	628 (38.0)	406 (24.9)	386 (33.7)	247 (22.5)
Rented private/other	291 (17.6)	263 (16.1)	177 (15.5)	172 (15.7)
	P < 0.001		P < 0.001	
Maternal alcohol[a]				
Never	870 (50.9)	827 (49.6)	602 (51.7)	566 (51.5)
<1 glass per week	563 (32.9)	530 (31.8)	376 (32.3)	348 (31.6)
1+ glasses per week	277 (16.2)	311 (18.7)	186 (16.0)	186 (16.9)
	P = 0.170		P = 0.830	
Maternal age (yr)[b]	25.8 (5.1)	26.8 (5.1)	26.1 (5.1)	26.8 (5.0)
	P < 0.001		P = 0.003	
Maternal birthweight (kg)[b]	3.12 (0.67)	3.32 (0.63)	3.20 (0.65)	3.22 (0.67)
	P<0.001		P=0.570	

[a] n(%); [b] mean (SD); MGM maternal grandmother; PGM paternal grandmother; + smoked in pregnancy; - did not smoke in pregnancy

For peer review only

Comparison of data concerning the potential confounders [Table 1] indicates that if either grandmother had smoked prenatally, then the smoking study mother herself was more likely to be a heavy smoker, to have had lower educational attainment and to be younger; in addition the family was more likely to be living in rented public housing. Not surprisingly, the women who had been exposed in utero (i.e. MGM+) had considerably lower mean birth weight themselves (by 199g) than those not exposed (MGM-). There was no difference in prevalence of smoking by the study father if his own mother had smoked during pregnancy.

Table 2 compares the birth measurements of study children born to parents who had been exposed to smoking *in utero*. It can be seen that for the women who had themselves been exposed *in utero*, there was just one statistically significant unadjusted association in their progeny (a lower birth weight for girls), but that this was no longer significant upon adjustment. For paternal *in utero* exposure, however, there were several unadjusted associations [with girls' birth weight and birth length, and with boys' birth length and head circumference]. On adjustment, the association with head circumference remained with a 0.35cm reduction [95% CI -0.57, -0.14] for boys [P = 0.001], but the association for girls was quite different: +0.08 [95%CI -0.11, +0.28]; (P for interaction = 0.006).

For peer review only

Table 2. Mean difference [(P value)] [95% CI] in birth measurements of children born to smoking mothers, comparing those where the child's grandmother had smoked with those who had not

	MGM+ M+ v. MGM- M+		PGM+ M+ v. PGM- M+	
	Unadjusted	Adjusted[a]	Unadjusted	Adjusted[a]
**BIRTHWEIGHT (g)**				
Boy	-13 (0.65)	-29 (0.24)	-55 (0.11)	-50 (0.074)
	[-69, +43]	[-77, +19]	[-123, +13]	[-104, +5]
Girl	**-63 (0.022)**	-31 (0.22)	**-88 (0.010)**	-11 (0.28)
	**[-116, -9]**	[-81, +18]	**[-155, -22]**	[-67, +45]
**BIRTH LENGTH (cm x100)**				
Boy	+8 (0.59)	-0 (1.00)	**-37 (0.035)**	-29 (0.070)
	[-20, +36]	[-28, +28]	**[-72, -3]**	[-61, +3]
Girl	-11 (0.44)	+7 (0.59)	**-37 (0.037)**	+1 (0.96)
	[-39, +17]	[-20, +35]	**[-71, -2]**	[-31, +33]
**HEAD CIRCUMFERENCE (cm x100)**				
Boy	+4 (0.66)	-3 (0.74)	**-35 (0.003)**	**-35 (0.001)**
	[-14, +23]	[-22, +16]	**[-59, -12]**	**[-57, -14]**
Girl	-9 (0.28)	-3 (0.76)	-17 (0.107)	+8 (0.39)
	[-27, +8]	[-19, +14]	[-39, +4]	[-11, +28]
**BMI ((kg/m^2)*10)**				
Boy	-0.3 (0.73)	-0.8 (0.37)	-1.2 (0.24)	-1.0 (0.31)
	[-1.9, +1.3]	[-2.5, +0.9]	[-3.1, +0.8]	[-2.9, +0.9]
Girl	-1.8 (0.41)	-1.3 (0.15)	-2.1 (0.056)	-0.6 (0.57)
	[-3.5, -0.1]	[-3.1, +0.5]	[-4.2, +0.1]	[-2.6, +1.5]

[a]Adjusted for maternal parity, maternal education, partner smoked in pregnancy, gestational length at birth of study child and the amount the mother smoked

[N.B. the data for birth length and head circumference are given in cm x 100 so as to aid viewing]

For peer review only

Sensitivity analyses

The analyses were repeated for primiparae and multiparae separately [Supplementary Tables 1 and 2]. The only significant association that remained after adjustment concerned the head circumference of the study sons. The effect sizes were similar for each parity group: for primiparae the effect size was -0.34 [95%CI -0.66, -0.02]cm, P = 0.036; for multiparae the adjusted effect size was similar at -0.35 [95% CI -0.64, -0.06]cm, P= 0.017. Again there were significant interactions with the sex of the child.

Since this association with head circumference was consistent but unexpected, and since there is evidence that birth head circumference is associated with childhood IQ,[30] we used the same study methodology to assess whether a similar association was apparent between paternal prenatal exposure and childhood IQ. Table 3 demonstrates that there was indeed a reduction in adjusted mean IQ of 2.90 points [95% CI -5.72, -0.08] (P = 0.044) for sons of exposed fathers, but no such association for daughters, although the interaction with sex was not statistically significant. Full scale IQ is made up of the sum of two components [performance IQ and verbal IQ] that are, in general, known to have different genetic and environmental components.[31] We therefore have analysed the data to assess whether the associations with paternal grandmothers' smoking during pregnancy are associated with one of these components in particular. We found that paternal exposure *in utero* had a greater effect on his son's verbal IQ [mean adjusted difference -3.65 points; 95% CI -6.60, -0.70], but with little difference in performance IQ [mean -1.40 [95% CI -4.39, +1.60] points.

For peer review only

Table 3. Mean difference [95% CI] in birth measurements of children born to smoking mothers, comparing those where the child's grandmother had smoked with those who had not.

	MGM+M+ v. MGM-M+		PGM+M+ v. PGM-M+	
	Unadjusted	Adjusted[a]	Unadjusted	Adjusted[a]
**Total IQ**				
Boys	**-3.87**	-2.45	**-4.00**	**-2.90**
95% CI	**[-6.34, -1.40]**	[-4.96, +0.07]	**[-6.92, -1.08]**	**[-5.72, -0.08]**
P value	**0.002**	0.057	**0.007**	**0.044**
Number	694	612	507	482
Girls	**-2.50**	-0.40	**-3.03**	-1.36
95% CI	**[-4.90, -0.11]**	[-2.85, +2.05]	**[-5.78, -0.28]**	[-4.07, +1.36]
P value	**0.041**	0.749	**0.031**	0.327
Number	617	551	456	436
**Performance IQ**				
Boys	**-2.64**	-1.48	-2.44	-1.40
95% CI	**[-5.20, -0.08]**	[-4.20, +1.24]	[-5.40, +0.50]	[-4.39, +1.60]
P value	**0.043**	0.287	0.104	0.360
Number	698	616	510	485
Girls	-2.46	-0.50	**-3.03**	-1.74
95% CI	[-5.01, +0.09]	[-3.19, +2.19]	**[-5.88, -0.18]**	[-4.69, +1.20]
P value	0.059	0.716	**0.037**	0.245
N	619	552	457	436
**Verbal IQ**				
Boys	**-3.75**	-2.40	**-4.73**	**-3.65**
95% CI	**[-6.30, -1.21]**	[-5.00, +1.20]	**[-7.81, -1.66]**	**[-6.60, -0.70]**
P value	**0.004**	0.070	**0.003**	**0.015**
Number	697	615	509	484
Girls	-1.98	-0.15	-2.48	-0.81
95% CI	[-4.37, +0.42]	[-2.60, +2.30]	[-5.28, +0.32]	[-3.54, +1.92]
P value	0.106	0.906	0.082	0.561
Number	617	551	456	436

[a]Adjusted for maternal education, parity, partner smoked in pregnancy, gestational length at birth of study child and the amount the mother smoked.

For peer review only

DISCUSSION

We investigated whether the parents' exposure *in utero* to their own mothers' smoking was associated with differences in fetal growth among women who smoked in pregnancy, and showed an association between paternal *in utero* exposure and a reduced head circumference in his sons, but not in his daughters. This was an unexpected finding. A series of sensitivity analyses showed the effect to be almost identical in children born to primiparae and to those born to multiparae. We assessed whether there was confirmatory evidence of an impact on brain size by looking at the IQ of the children. We found a significant reduction in total IQ in 8-year-old boys (but not girls) whose paternal grandmother smoked during the pregnancy resulting in the study child's father. The IQ effect size was similar in both parity groups and was still present when birth head circumference was taken into account [data not shown]. We showed a stronger association with verbal IQ than performance IQ. To our knowledge there have been no previous studies that have considered any effects of paternal exposure to smoking *in utero* on his offspring.

Strengths and limitations

There are a number of limitations to this study: (i) Details of smoking of parents and grandparents depend on parental self-report – however there is considerable information to indicate that adults are unlikely to lie about smoking habits, especially when using anonymised self-completion questionnaires;[32] here we have shown that the mean birth weight of the study mothers who had reported that their own mothers had smoked when they were *in utero* was 199g lower than that of those who had reported that their mother did not smoke at that time, which was about the expected order of difference if the mothers had reported accurately; (ii) although the amount the parents smoked was reported, there was no

For peer review only - http://bmjopen.bmj.com/site/about/guidelines.xhtml

estimate requested for the amount smoked by the grandmothers when pregnant with the study parent – this may have been associated with the outcome, but it is difficult to postulate how such effects might differ between the sexes of the study children; (iii) although the ALSPAC study is large, the numbers of women who smoked throughout pregnancy and for whom details are available on the grandmothers' smoking are reduced and consequently the statistical power is relatively low. Among the strengths of this study are the following: (a) it tested a prior hypothesis that early life exposures can have phenotypic effects down the paternal line with sex-specific outcomes; (b) the information on grandparental and parental smoking was collected prior to the birth of the study child, and consequently cannot have been biased by knowledge of fetal size; (c) birth length and head circumference were ascertained by trained measurers using standard techniques, as opposed to the generally inaccurate methods used in most delivery units; (d) IQ was measured using standard methodology by trained psychologists; (e) the study was based on a relatively large population sample, and results are therefore likely to be generalisable.

Meaning of the study

Our parallel study of non-smoking mothers looked at the effect of parental exposure *in utero*; we found the sons were larger at birth (both in regard to birth weight and birth BMI) if the maternal grandmother had smoked in the pregnancy that resulted in the study mother. There was no discernible effect of paternal prenatal exposure on the study child's birth weight or BMI; however there was a slight *increase* in head circumference among the boys born to fathers who had been exposed *in utero* [mean difference +0.08 cm; 95% CI -0.03, +0.19].[8]

For peer review only

Attributing the smaller head circumference in boys of smoking mothers to the prenatal exposure of the father through his own mother's smoking, raises the question of possible mechanisms. How might the information be transmitted via his sperm or in some other way? As we noted in the introduction to this paper there is increasing evidence that exposures, especially in early life, can lead to enduring changes in the epigenome that, in turn, can modify gene expression. Whilst transgenerational epigenetic inheritance remains controversial, at least in humans,[33] the phenomenon of genomic imprinting establishes the principle that epigenetic marks such as DNA methylation placed in one generation can influence gene expression in the next. One such imprinted gene is the Insulin Growth Factor 2 (*IGF2*) which is expressed only from the paternally-derived chromosome 11, the maternal copy being epigenetically silenced. *IGF2* encodes an endocrine and autocrine/paracrine acting factor important in directing growth during prenatal development.[34,35] Maternal smoking has been shown to be associated with a 5% higher DNA methylation level at the *IGF2* DMR (differentially methylated region) in the newborn infant,[5] and interestingly in the context of our study, this methylation shift is specific to male offspring. Thus it is possible that the study father's *IGF2* DMR had been epigenetically modified (including in his fetal testes) by his mother's smoking throughout pregnancy. Furthermore it is plausible that this epigenetic state could be transmitted via his sperm to the study offspring. Imprinted gene regions tend to escape the usual widespread erasure of DNA methylation from the paternally-derived genome in the pre-implantation embryo soon after fertilisation.[36] In support of paternal effects generally, there is a report of hypomethylation at the *IGF2* DMR in umbilical cord blood being associated with paternal obesity suggesting a preconceptional impact of the obesity (and/or exposures related to it) on the reprogramming of imprint marks during spermatogenesis.[37]

For peer review only

In conclusion

When the mother is a smoker, we found no effect of her own tobacco exposure *in utero* on the fetal growth of her children. However, when the mother is a smoker, paternal exposure *in utero* is associated with a reduced head circumference at birth and IQ at 8 years in sons, but not daughters. We had no prior hypothesis that head circumference would be associated, particularly among sons, so these results must be considered as hypothesis generating, and require testing in further longitudinal data sets.

For peer review only

Acknowledgements

We are extremely grateful to all the families who took part in this study, the midwives for their help in recruiting them, and the whole ALSPAC team, which includes interviewers, computer and laboratory technicians, clerical workers, research scientists, volunteers, managers, receptionists and nurses.

Contributors: MP and JG had the idea; KN, SG and LLM carried out the statistical analyses; JG and MP wrote the first draft and all authors contributed to the final manuscript.

Funding: The UK Medical Research Council (MRC), the Wellcome Trust and the University of Bristol currently provide core support for ALSPAC. The statistical analyses for this paper were undertaken with funding from the Medical Research Council (grant no. G1100226). The funders had no role in study design, data collection, statistical analysis, decision to publish, or preparation of the manuscript.

Competing interests: The authors have no competing interests.

Ethics approval

Ethical approval for the ALSPAC study was obtained from the ALSPAC Law and Ethics Committee and the three Avon-based Local Research Ethics Committees: Bristol and Weston Health Authority: E1808 Children of the Nineties: Avon Longitudinal Study of Pregnancy and Childhood (ALSPAC) (28th November 1989). Southmead Health Authority: 49/89 Children of the Nineties - "ALSPAC" (5th April 1990). Frenchay Health Authority: 90/8 Children of the Nineties. (28th June 1990). Written consent was obtained for all assays of biological samples. Ethics Committees considered voluntarily returned postal questionnaires as implied consent.

For peer review only

Data sharing: ALSPAC is committed to share data with bona fide researchers. See the study website for the conditions of use and access procedures: http://www.bristol.ac.uk/alspac/researchers/

For peer review only

References

1. Barker DJ. In utero programming of chronic disease. *Clin Sci* 1998;**95**:115-128.

2. Gluckman PD, Hanson MA, Buklijas T, et al. Epigenetic mechanisms that underpin metabolic and cardiovascular disease. *Nat Rev Endocrinol* 2009;**5**:401- 408. doi:10.1038/nrendo.2009.102

3. Relton C, Davey-Smith G, Ozanne S. Developmental Epigenetic Programming in Diabetes and Obesity. In: Jirtle R, Tyson E, eds. Environmental Epigenomics in Health and Disease; Epigenetics and Complex Diseases. Berlin Heidelberg: Springer 2013:235-253.

4. Breton CV, Byun HM, Wenten M, et al. Prenatal tobacco smoke exposure affects global and gene-specific DNA methylation. *Am J Respir Crit Care Med* 2009;**180**:462-467. Doi. 10.1164/rccm.200901-0135OC

5. Murphy SK, Adigun A, Huang Z, et al. Gender-specific methylation differences in relation to prenatal exposure to cigarette smoke. *Gene* 2012;**15**:36-43. doi.org/10.1016/j.gene.2011.11.062.

6. Joubert BR, Håberg SE, Nilsen RM, et al. 450K epigenome-wide scan identifies differential DNA methylation in newborns related to maternal smoking during pregnancy. *Environ Health Perspect* 2012;**120**:1425-1431. 10.1289/ehp.1205412.

7. Lee KKW, Pausova Z. Cigarette smoking and DNA methylation. *Front Genet* 2013;**4**:132. doi: 10.3389/fgene.2013.00132

8. Miller LL, Pembrey M, Davey Smith G, et al. Is the growth of the fetus of a non-smoking mother influenced by the smoking of either grandmother while pregnant? *PLoS ONE* 2014; 9(2): e86781. doi:10.1371/journal.pone.0086781.

For peer review only

9. Davey Smith G. Assessing intrauterine influences on offspring outcomes: Can epidemiological studies yield robust outcomes? *Basic Clin Pharmacol Toxicol* 2008;**102**:245-256. DOI: 10.1111/j.1742-7843.2007.00191.x

10. Macdonald-Wallis C, Tobias JH, Davey Smith G, et al. Parental smoking during pregnancy and offspring bone mass at age 10 years: findings from a prospective birth cohort. *Osteoporos Int* 2011; **22**:1809-1819. Doi: 10.1007/s00198-010-1415-y

11. Bygren LO, Kaati G, Edvinsson S. Longevity determined by ancestors' over nutrition during their slow growth period. *Acta Biotheor* 2001;**49**:53-59. Doi. 10.1023/A:1010241825519

12. Kaati G, Bygren LO, Edvinsson S. Cardiovascular and diabetes mortality determined by nutrition during parents' and grandparents' slow growth period. *Eur J Hum Genet* 2002;**10**:682-688. DOI:10.1038/sj.ejhg.5200859

13. Pembrey ME, Bygren LO, Kaati G et al. Sex-specific, male-line transgenerational responses in humans. *Eur J Hum Genet* 2006;**14**:159–166. doi:10.1038/sj.ejhg.5201538

14. Anway MD, Cupp AS, Uzumcu M, et al. Epigenetic transgenerational actions of endocrine disruptors and male fertility. *Science* 2005;**308**:1466-1469. DOI: 10.1126/science.1108190

15. Franklin TB, Russig H, Weiss IC et al. Epigenetic transmission of the impact of early stress across generations. *Biol Psychiatr* 2010;**68**:408-415.doi10.1016/j.biopsych.2010.05.036

16. Ng SF, Lin RC, Laybutt DR et al. Chronic high-fat diet in fathers programs β-cell dysfunction in female rat offspring. *Nature* 2010;**467**:963-966. doi:10.1038/nature09491

17. Zeybel M, Hardy T, Wong YK, et al. Multigenerational epigenetic adaptation of the hepatic wound-healing response. *Nat Med* 2012;**18**:1369–1377. doi:10.1038/nm.2893

For peer review only

18. Dunn GA, Bale TL. Maternal high-fat diet effects on third-generation female body size via the paternal lineage. *Endocrinol* 2011;**152**:2228-2236. Doi:10.1210/en.2010.1461

19. Burdge GC, Slater-Jefferies J, Torrens C, et al. Dietary protein restriction of pregnant rats in the F0 generation induces altered methylation of hepatic gene promoters in the adult male offspring in the F1 and F2 generations. *Br J Nutr* 2007;**97**:435-439. Doi 10.1017/S0007114507352392

20. Carone BR, Fauquier L, Habib N, et al. Paternally induced transgenerational environmental reprogramming of metabolic gene expression in mammals. *Cell* 2010;**143**:1084-1096. Doi:10.1016/j.cell.2010.12.008

21. Dias BG, Ressler KJ. Parental olfactory experience influences behavior and neural structure in subsequent generations. *Nat Neurosci* 2014;**17**:89-96. Doi: doi:10.1038/nn.3594

22. Daxinger L, Whitelaw E. Understanding transgenerational epigenetic inheritance via the gametes in mammals. *Nat Rev Genet* 2012;**31**:153-162. doi:10.1038/nrg3188

23. Misra DP, Astone N, Lynch CD. Maternal smoking and birth weight: interaction with parity and mother's own in utero exposure to smoking. *Epidemiol* 2005;**16**:288–293. doi: 10.1097/01.ede.0000158198.59544.cf

24. Golding J, Pembrey M, Jones R. ALSPAC–the Avon Longitudinal Study of Parents and Children. I. Study methodology. *Paediatr Perinatal Epidemiol* 2001;**15**:74–87. DOI: 10.1046/j.1365-3016.2001.00325.x

25. Boyd A, Golding J, Macleod J, et al. Cohort Profile: The 'Children of the 90s'–the index offspring of the Avon Longitudinal Study of Parents and Children. *Int J Epidemiol* 2012;**42**:111-127. doi. 10.1093/ije/dys064

26. ALSPAC web: http://www.bristol.ac.uk/alspac/researchers/resources-available/data-details/questionnaires/. Accessed 2 December 2013.

view only

27. Cole TJ, Henson GL, Tremble JM, et al. Birthweight for length: ponderal index, body mass index or Benn index? *Ann Hum Biol* 1997;**24**:289–298. doi:10.1080/03014469700005032

28. Gale CR, O'Callaghan FJ, Bredow M, et al. The influence of head growth in fetal life, infancy, and childhood on intelligence at the ages of 4 and 8 years. *Pediatr* 2006;**118**:1486-1492. doi: 10.1542/peds.2005-2629

29. Wechsler D, Golombok S, Rust J. WISC-IIIUK Wechsler Intelligence Scale for Children. Sidcup, UK: Psychological Corp 1992.

30. Gunnell D, Miller LL, Rogers I, et al. Association of Insulin-like Growth Factor I and Insulin-like Growth Factor–Binding Protein-3 With Intelligence Quotient Among 8-to 9-Year-Old Children in the Avon Longitudinal Study of Parents and Children. *Pediatr* 2005; **116**:e681-e686. doi: 10.1542/peds.2004-2390

31. Dickens WT, Flynn JR. Heritability estimates versus large environmental effects: the IQ paradox resolved. *Psychol Rev* 2001;**108**:346. Doi:10.1037/0033-295X.108.2.346

32. Klebanoff MA, Levine RJ, Clemens JD, et al. Serum cotinine concentration and self-reported smoking during pregnancy. *Am J Epidemiol* 1998;**148**:259-262.

33. Grossniklaus U, Kelly B, Ferguson-Smith AC, et al. Transgenerational epigenetic inheritance: how important is it? *Nat Rev Genet* 2013;**14**:228-235. doi:10.1038/nrg3435

34. LeRoith D, Lowe WL. Growth factors. In: Melmed S, Conn PM, eds. Endocrinology: Basic and Clinical Principles, 2nd edition. Totowa, New Jersey: Humana Press 2005:85-91.

35. Demetriou C, Abu-Amero S, Thomas AC, et al. Paternally expressed, imprinted Insulin-1 like growth factor-2 in chorionic villi correlates significantly with birth weight. *PLoS ONE* 2014;doi:10.1371/journal.pone.0085454.

For peer review only

36. Seisenberger S, Peat JR, Hore TA, et al. Reprogramming DNA methylation in the mammalian life cycle: building and breaking epigenetic barriers. *Philos Trans R Soc Lond B Biol Sci* 2013;**368**: 20110330. doi: 10.1098/rstb.2011.0330.

37. Soubry A, Schildkraut JM, Murtha A, et al. Paternal obesity is associated with IGF2 hypomethylation in newborns: results from a Newborn Epigenetics Study (NEST) cohort. *BMC Med* 2013;29:doi:10.1186/1741-7015-11-29

For peer review only

Is the growth of the child of a smoking mother influenced by the father's prenatal exposure to tobacco? A hypothesis generating longitudinal study

Marcus Pembrey[abc], Kate Northstone[a], Steven Gregory[ab], Laura L Miller[a], Jean Golding[ab]

[a]School of Social and Community Medicine, [b]Centre for Child and Adolescent Health, University of Bristol, Bristol and [c] Institute of Child Health, University College London, London, UK

For peer review only

Key Words: ALSPAC, transgenerational effects, grandmothers' prenatal smoking, birth head circumference, IQ

Correspondence to:

Professor Jean Golding,
Centre for Child and Adolescent Health,
School of Social and Community Medicine,
Barley House,
Oakfield Grove
Bristol BS8 2BN,
UK

Email: jean.golding@bristol.ac.uk
Telephone: +44 (0)117 3310198 Fax: +44 (0)117 3313303

14.55.2.14 **Word Count: 3432**

2

Abstract

Objectives: Transgenerational effects of different environmental exposures is becoming of major interest, with rodent experiments focusing on epigenetic mechanisms. Previously we have shown that if the study mother is a non-smoker, there is increased mean birth weight, length and body mass index [BMI] in her sons if she herself had been exposed prenatally to her mother's smoking. The aim of this study was to determine whether the prenatal smoke exposure of either parent influenced the growth of the fetus of a smoking woman, and whether any effects were dependent on the fetal sex.

Design: Population based pre-birth cohort study.

Setting: Avon Longitudinal Study of Parents and Children.

Participants: Participants were residents of a geographic area with expected date of delivery between April 1991 and December 1992. Among pregnancies of mothers who smoked during pregnancy, data were available concerning maternal and paternal prenatal exposures to their own mother smoking for 3502 and 2354 respectively

Primary and secondary outcome measures:, Birth weight, length, BMI and head circumference.

Results: After controlling for confounders, there were no associations with birth weight, length or BMI. There was a strong adjusted association of birth head circumference among boys whose fathers had been exposed prenatally [mean difference -0.35cm; 95%CI -0.57,-0.14; P=0.001]. There was no such association with girls (interaction: P=0.006). Similar associations were found when primiparae and multiparae were analysed separately. In order to determine whether this was reflected in child development, we examined the relationships with IQ; we found that the boys born to exposed fathers had lower IQ scores on average, and that this was particularly due to the verbal component [mean difference in verbal IQ: -3.65; 95%CI -6.60,-0.70 points].

For peer review only

3

Conclusions: Head size differences concerning paternal fetal exposure to smoking were unexpected and, as such, should be regarded as hypothesis generating.

For peer review only

4

Strengths and limitations of this study

- This study is the first to examine the sex-specific fetal effects of parental prenatal exposure to cigarette smoking when the mother herself smoked during pregnancy.
- Data were collected on a population sample who completed questionnaires blind to the study hypotheses.
- Birth measurements were undertaken using trained staff with repeated validation.
- A variety of sensitivity analyses were undertaken, including separate analyses of primiparae and multiparae, as well as of follow-up of the offspring to determine whether the decrement in birth head circumference was reflected in childhood measurement of IQ. All were in accord with the initial finding.
- The limitation of the study is the failure to obtain comparable data to confirm or negate the study findings.

Funding: The UK Medical Research Council (www.mrc.ac.uk), the Wellcome Trust (www.Wellcome.ac.uk) and the University of Bristol (www.bristol.ac.uk) currently provide core support for ALSPAC. The statistical analyses for this project were undertaken with funding from the Medical Research Council [grant no. G1100226]. The funders had no role in study design, data collection, statistical analysis, decision to publish, or preparation of the manuscript.

Competing interests: The authors have no competing interests.

For peer review only

INTRODUCTION

Fetal programming via the mother's nutrition and other aspects of her environment is well-recognised as a contributor to adult morbidity and mortality[1] and some of these enduring effects are likely to be mediated by epigenetic mechanisms.[2,3] Studies have shown specific DNA methylation patterns in children whose mothers had smoked during pregnancy.[4-7] However, there have been few pre-conceptional transgenerational studies relating the fetal environment of either parent to the birth outcomes of their own children.

In an earlier study of non-smoking mothers, we found an increase in the birth weight and birth body mass index [BMI] of her sons if she had been exposed *in utero* to her own mothers' smoking, but there was no such effect if the study father had been exposed *in utero*.[8] This lack of paternal influence from his own intrauterine exposure was not unexpected. Indeed, it has been proposed that the paternal line can act as a form of control in studies of maternal effects.[9,10] However, this was not our reason for analysing potential paternal exposure transgenerational effects in our earlier paper[8] or in the present analysis of smoking mothers. They were instigated by studies from Sweden based on samples of individuals born in the town of Överkalix. Their longevity and other health outcomes were linked to detailed historical records of harvests experienced by their ancestors. Although most of the emphasis in the Överkalix study was concerned with exposures in mid-childhood[11,12] the studies of three cohorts pooled together have demonstrated effects of exposures of the paternal grandmother [PGM] prenatally to times of very poor harvests on significantly increased mortality of her granddaughters but not her grandsons.[13] Thus, the presumed effect is from the *in utero* exposure of the paternal grandmother to her son and subsequently to his daughter. Such transgenerational effects are now well supported by rodent experiments showing male-line transmissions and often demonstrating sex-specific

For peer review only

transmission on outcomes,[14-17] some focusing on imprinted gene expression in descendants[18] and others on associated epigenetic changes,[19-21] although no transgenerational signal itself has been clearly defined.[22]

Our earlier transgenerational study of intrauterine exposure of non-smoking mothers did not consider relationships with fetal growth if the study mother was also a smoker.[8] Here we use the same cohort, the Avon Longitudinal Study of Parents and Children [ALSPAC], to investigate the fetal growth of offspring of smoking mothers only – comparing the offspring of mothers and fathers who were themselves exposed to cigarette smoke *in utero* with those who were not exposed in this way. The only study that we are aware of that has looked at an aspect of this question compared the birth weight of the grandchildren comparing those born to mothers who smoked according to whether they themselves had been exposed to their own mothers' smoking *in utero*.[23] Altogether they reported a decrease of 70g if the grandmother had also smoked during pregnancy. The authors did not assess whether this difference was merely a function of variation in the amount smoked by the study mother. Nor did they assess whether there was any effect discernible with the prenatal smoke exposure of the study father, or whether there was any difference between the effects depending on the sex of the offspring.

The current study was therefore carried out to assess whether there is indeed a reduction in the growth of the fetus of a smoking mother if her own mother smoked, and/or whether exposure of the father in utero has any effect on the growth of the child of the smoking mother. In line with the evidence of the accumulating transgenerational human and animal data outlined above, we hypothesise that any effects will differ between boy and girl infants.

For peer review only

METHODS

Ethics statement

Ethical approval for the ALSPAC study was obtained from the ALSPAC Law and Ethics Committee and the three Avon-based Local Research Ethics Committees: Bristol and Weston Health Authority: E1808 Children of the Nineties: Avon Longitudinal Study of Pregnancy and Childhood (ALSPAC) (28th November 1989). Southmead Health Authority: 49/89 Children of the Nineties – "ALSPAC" (5th April 1990). Frenchay Health Authority: 90/8 Children of the Nineties. (28th June 1990). Written consent was obtained for all assays of biological samples. Ethics Committees considered voluntarily returned postal questionnaires as implied consent.

Study sample

The data used in these analyses were collected as part of the Avon Longitudinal Study of Parents and Children (ALSPAC), which was designed to assess the ways in which the environment interacts with the genotype to influence health and development.[24] Pregnant women, resident in the study area in south-west England with an expected date of delivery between 1st April 1991 and 31st December 1992, were invited to take part. About 80% of the eligible population did so.[25]

Information collected from the study parents during their study pregnancy included details of the maternal and paternal grandparents. Figure 1 illustrates the two pathways of possible influence of parental prenatal exposure to cigarette smoke on the study child that we investigate in this paper.

Figure 1. Diagram of intergenerational linkage, where MGM = maternal grandmother; PGM = paternal grandmother

For peer review only

The exposures

The women and their partners were sent a number of questionnaires during pregnancy.[26] These elicited information on their current smoking habits and those of their parents (i.e. the study grandparents). If they reported that their mothers had smoked, they were asked whether their mothers had smoked when expecting them – and, if so, were given the responses: yes / no / don't know from which to select. Thus the parents who replied 'don't know', had a mother who smoked but the parent was unsure whether she had smoked during her pregnancy. We have analysed these data in two ways: (a) assuming that all these women did smoke during pregnancy, and (b) omitting the 'don't knows' from the analyses and only analysing those definitely reported as smoking during the study pregnancy [this we have treated as a sensitivity analysis]. All mothers who themselves did not smoke during the study pregnancy were excluded from these analyses. Consequently we compared two groups of grandchildren: those whose grandmothers had smoked during the pregnancy resulting in their parent and whose mothers had also smoked during the pregnancy that resulted in the study child [MGM+M+ and PGM+M+] with those whose grandmothers had not smoked [MGM-M+ and PGM-M+] respectively. In these analyses all study mothers smoked during pregnancy. Analyses of fetal growth measures took account of the highest amount smoked by the mother during the study pregnancy, grouped as <10; 10-19; 20+ per day.

Possible confounders

Other data used in the analyses include the study mother's parity (as ascertained from the maternal report of previous pregnancies resulting in either a live- or still-birth, and coded as 0; 1+); gestation (completed weeks: 39+; 37-38; ≤36); mother's partner smoking at the start of pregnancy (primarily reported by the partner, but maternal report was used if the partner report was missing: yes; no); maternal age at the birth of the child (continuous);

For peer review only

housing tenure as a measure of socioeconomic background (owned or mortgaged; rented public housing; all other), maternal education (highest level of educational attainment – in five levels of increasing achievement), maternal alcohol consumption when the mother first felt the baby move (not at all; <1 glass per week; and one or more glasses per week).

Outcome measures

At delivery the baby was weighed to the nearest gram; ALSPAC study staff visited the two main delivery hospitals each day and measured the crown-heel length and head circumference of available infants in a standardised manner.[24] Body mass index (BMI) was calculated as birth weight/length^2 (g/m^2). In this study we have used BMI, rather than ponderal index (PI) as our measure of adiposity at birth; although it is traditional to use PI at birth, there is little literature to justify this. It has been suggested that the criteria used to choose whether to use PI or BMI should be a measure that is independent of length.[27] We have assessed which of the two measures is independent of length at each gestation among ALSPAC births and found that BMI satisfies the independence requirement more closely than PI.[8]

Pilot studies before the start of the ALSPAC study had demonstrated that it was usually the student midwife who was given the task of measuring the circumference of the baby's head; she tended to have had little or no training and the measurements made were grossly inaccurate. For this study we only used measurements that were made by our own staff, after detailed training and with repeated validation over time. ~~Detailed studies with accurate data to test the hypothesis raised by the results of this study are needed.~~

Statistical analyses

For peer review only

Multivariable linear regression models assessed the study children's adjusted mean birth weight, crown-heel length, head circumference and BMI by parental prenatal smoking exposure. All models were adjusted for parity, maternal education, amount mother smoked, paternal smoking in pregnancy and gestation with MGM-M+ (and PGM-M+) as the reference categories. Additional models adjusted for maternal age, housing tenure and maternal alcohol use as well as maternal birth weight.

Sensitivity analyses

In order to determine consistency of the findings, separate analyses were undertaken for primiparae and multiparae. In order to determine whether the head circumference results were biologically meaningful, we also used the fact that reduced head circumference is associated with lower levels of childhood IQ.[28] Childhood IQ was assessed by trained psychologists at age 8 years (56·2% of eligible children attended), with an abbreviated form of the Wechsler Intelligence Scale for Children (WISC-III).[29] This abbreviated form has been shown to be a valid method for use in research studies.[30]

RESULTS

In all, there were 3502 births to smoking mothers for whom data were available as to whether their own mothers had smoked when expecting them [Table 1]. Approximately half had such a history. Fewer women had information about the prenatal exposures of the father of their study child [n= 2354], but again exposure was approximately 50:50.

For peer review only

Table 1. The study sample of mothers who smoked during pregnancy

	MGM+	MGM-	PGM+	PGM-
**No. in study**	**1781**	**1721**	**1209**	**1145**
Maternal smoking in pregnancy (cigarettes per day)[a]				
<10	500 (28.0)	699 (40.6)	402 (33.3)	460 (40.2)
10-19	811 (45.6)	698 (40.6)	524 (43.3)	475 (41.5)
20+	469 (26.4)	324 (18.8)	283 (23.4)	209 (18.3)
	P < 0.001		P <0.001	
Parity[a]				
0	747 (43.3)	779 (46.1)	511 (43.3)	537 (47.8)
1+	980 (56.8)	910 (53.9)	668 (56.7)	586 (52.2)
	P = 0.092		P = 0.031	
Maternal education level[a]				
CSE or less	598 (39.6)	399 (26.3)	362 (33.8)	284 (27.6)
Vocational	195 (12.9)	184 (12.2)	144 (13.5)	126 (12.3)
O Level	493 (32.6)	548 (36.2)	367 (34.3)	357 (34.7)
A Level	181 (12.0)	291 (19.2)	156 (14.6)	195 (19.0)
Degree	45 (3.0)	93 (6.1)	41 (3.8)	66 (6.4)
	P<0.001		P<0.001	
Gestation(weeks)[a]				
39+	1328 (75.1)	1279 (74.6)	860 (71.5)	868 (75.9)
37/38	305 (17.3)	312 (18.2)	249 (20.7)	197 (17.2)
<37	135 (7.6)	123 (7.2)	94 (7.8)	78 (6.8)
	P = 0.070		P = 0.048	
Partner smoking[a]				
No	478 (29.7)	487 (30.3)	341 (28.3)	344 (30.0)
Yes	1130 (70.3)	1120 (69.7)	863 (71.7)	801 (70.0)
	P = 0.072		P = 0.360	
Housing tenure[a]				
Owned/mortgaged	736 (44.5)	965 (59.1)	583 (50.9)	677 (61.8)
nted public	628 (38.0)	406 (24.9)	386 (33.7)	247 (22.5)
Rented private/other	291 (17.6)	263 (16.1)	177 (15.5)	172 (15.7)
	P < 0.001		P < 0.001	
Maternal alcohol[a]				
Never	870 (50.9)	827 (49.6)	602 (51.7)	566 (51.5)
<1 glass per week	563 (32.9)	530 (31.8)	376 (32.3)	348 (31.6)
1+ glasses per week	277 (16.2)	311 (18.7)	186 (16.0)	186 (16.9)
	P = 0.170		P = 0.830	
Maternal age (yr)[b]	25.8 (5.1)	26.8 (5.1)	26.1 (5.1)	26.8 (5.0)
	P < 0.001		P = 0.003	
Maternal birthweight (kg)[b]	3.12 (0.67)	3.32 (0.63)	3.20 (0.65)	3.22 (0.67)
	P<0.001		P=0.570	

[a] n(%); [b] mean (SD); MGM maternal grandmother; PGM paternal grandmother; + smoked in pregnancy; - did not smoke in pregnancy

For peer review only

Comparison of data concerning the potential confounders [Table 1] indicates that if either grandmother had smoked prenatally, then the smoking study mother herself was more likely to be a heavy smoker, to have had lower educational attainment and to be younger; in addition the family was more likely to be living in rented public housing. Not surprisingly, the women who had been exposed in utero (i.e. MGM+) had considerably lower mean birth weight themselves (by 199g) than those not exposed (MGM-). There was no difference in prevalence of smoking by the study father if his own mother had smoked during pregnancy.

Table 2 compares the birth measurements of study children born to parents who had been exposed to smoking *in utero.* It can be seen that for the women who had themselves been exposed *in utero*, there was just one statistically significant unadjusted association in their progeny (a lower birth weight for girls), but that this was no longer significant upon adjustment. For paternal *in utero* exposure, however, there were several unadjusted associations [with girls' birth weight and birth length, and with boys' birth length and head circumference]. On adjustment, the association with head circumference remained with a 0.35cm reduction [95% CI -0.57, -0.14] for boys [P = 0.001], but the association for girls was quite different: +0.08 [95%CI -0.11, +0.28]; (P for interaction = 0.006).

For peer review only

13

Table 2. Mean difference $^{(P\ value)}$ [95% CI] in birth measurements of children born to smoking mothers, comparing those where the child's grandmother had smoked with those who had not

	MGM+ M+ v. MGM- M+		PGM+ M+ v. PGM- M+	
	Unadjusted	Adjusted[a]	Unadjusted	Adjusted[a]
**BIRTHWEIGHT (g)**				
Boy	-13 (0.65)	-29 (0.24)	-55 (0.11)	-50 (0.074)
	[-69, +43]	[-77, +19]	[-123, +13]	[-104, +5]
Girl	**-63 (0.022)**	-31 (0.22)	**-88 (0.010)**	-11 (0.28)
	**[-116, -9]**	[-81, +18]	**[-155, -22]**	[-67, +45]
**BIRTH LENGTH (cm x100)**				
Boy	+8 (0.59)	-0 (1.00)	**-37 (0.035)**	-29 (0.070)
	[-20, +36]	[-28, +28]	**[-72, -3]**	[-61, +3]
Girl	-11 (0.44)	+7 (0.59)	**-37 (0.037)**	+1 (0.96)
	[-39, +17]	[-20, +35]	**[-71, -2]**	[-31, +33]
**HEAD CIRCUMFERENCE (cm x100)**				
Boy	+4 (0.66)	-3 (0.74)	**-35 (0.003)**	**-35 (0.001)**
	[-14, +23]	[-22, +16]	**[-59, -12]**	**[-57, -14]**
Girl	-9 (0.28)	-3 (0.76)	-17 (0.107)	+8 (0.39)
	[-27, +8]	[-19, +14]	[-39, +4]	[-11, +28]
**BMI ((kg/m^2)*10)**				
Boy	-0.3 (0.73)	-0.8 (0.37)	-1.2 (0.24)	-1.0 (0.31)
	[-1.9, +1.3]	[-2.5, +0.9]	[-3.1, +0.8]	[-2.9, +0.9]
Girl	-1.8 (0.41)	-1.3 (0.15)	-2.1 (0.056)	-0.6 (0.57)
	[-3.5, -0.1]	[-3.1, +0.5]	[-4.2, +0.1]	[-2.6, +1.5]

[a]Adjusted for maternal parity, maternal education, partner smoked in pregnancy, gestational length at birth of study child and the amount the mother smoked

[N.B. the data for birth length and head circumference are given in cm x 100 so as to aid viewing]

For peer review only

Sensitivity analyses

The analyses were repeated for primiparae and multiparae separately [Supplementary Tables 1 and 2]. The only significant association that remained after adjustment concerned the head circumference of the study sons. The effect sizes were similar for each parity group: for primiparae the effect size was -0.34 [95%CI -0.66, -0.02]cm, P = 0.036; for multiparae the adjusted effect size was similar at -0.35 [95% CI -0.64, -0.06]cm, P= 0.017. Again there were significant interactions with the sex of the child.

Since this association with head circumference was consistent but unexpected, and since there is evidence that birth head circumference is associated with childhood IQ,[30] we used the same study methodology to assess whether a similar association was apparent between paternal prenatal exposure and childhood IQ. Table 3 demonstrates that there was indeed a reduction in adjusted mean IQ of 2.90 points [95% CI -5.72, -0.08] (P = 0.044) for sons of exposed fathers, but no such association for daughters, although the interaction with sex was not statistically significant. Full scale IQ is made up of the sum of two components [performance IQ and verbal IQ] that are, in general, known to have different genetic and environmental components.[31] We therefore have analysed the data to assess whether the associations with paternal grandmothers' smoking during pregnancy are associated with one of these components in particular. We found that paternal exposure *in utero* had a greater effect on his son's verbal IQ [mean adjusted difference -3.65 points; 95% CI -6.60, -0.70], but with little difference in performance IQ [mean -1.40 [95% CI -4.39, +1.60] points.

For peer review only

Table 3. Mean difference [95% CI] in birth measurements of children born to smoking mothers, comparing those where the child's grandmother had smoked with those who had not.

	MGM+M+ v. MGM-M+		PGM+M+ v. PGM-M+	
	Unadjusted	Adjusted[a]	Unadjusted	Adjusted[a]
**Total IQ**				
Boys	**-3.87**	-2.45	**-4.00**	**-2.90**
95% CI	**[-6.34, -1.40]**	[-4.96, +0.07]	**[-6.92, -1.08]**	**[-5.72, -0.08]**
P value	**0.002**	0.057	**0.007**	**0.044**
Number	694	612	507	482
Girls	**-2.50**	-0.40	**-3.03**	-1.36
95% CI	**[-4.90, -0.11]**	[-2.85, +2.05]	**[-5.78, -0.28]**	[-4.07, +1.36]
P value	**0.041**	0.749	**0.031**	0.327
Number	617	551	456	436
**Performance IQ**				
Boys	**-2.64**	-1.48	-2.44	-1.40
95% CI	**[-5.20, -0.08]**	[-4.20, +1.24]	[-5.40, +0.50]	[-4.39, +1.60]
P value	**0.043**	0.287	0.104	0.360
Number	698	616	510	485
Girls	-2.46	-0.50	**-3.03**	-1.74
95% CI	[-5.01, +0.09]	[-3.19, +2.19]	**[-5.88, -0.18]**	[-4.69, +1.20]
P value	0.059	0.716	**0.037**	0.245
N	619	552	457	436
**Verbal IQ**				
Boys	**-3.75**	-2.40	**-4.73**	**-3.65**
95% CI	**[-6.30, -1.21]**	[-5.00, +1.20]	**[-7.81, -1.66]**	**[-6.60, -0.70]**
P value	**0.004**	0.070	**0.003**	**0.015**
Number	697	615	509	484
Girls	-1.98	-0.15	-2.48	-0.81
95% CI	[-4.37, +0.42]	[-2.60, +2.30]	[-5.28, +0.32]	[-3.54, +1.92]
P value	0.106	0.906	0.082	0.561
Number	617	551	456	436

[a]Adjusted for maternal education, parity, partner smoked in pregnancy, gestational length at birth of study child and the amount the mother smoked.

For peer review only

DISCUSSION

We investigated whether the parents' exposure *in utero* to their own mothers' smoking was associated with differences in fetal growth among women who smoked in pregnancy, and showed an association between paternal *in utero* exposure and a reduced head circumference in his sons, but not in his daughters. This was an unexpected finding. A series of sensitivity analyses showed the effect to be almost identical in children born to primiparae and to those born to multiparae. We assessed whether there was confirmatory evidence of an impact on brain size by looking at the IQ of the children. We found a significant reduction in total IQ in 8-year-old boys (but not girls) whose paternal grandmother smoked during the pregnancy resulting in the study child's father. The IQ effect size was similar in both parity groups and was still present when birth head circumference was taken into account [data not shown]. We showed a stronger association with verbal IQ than performance IQ. To our knowledge there have been no previous studies that have considered any effects of paternal exposure to smoking *in utero* on his offspring.

Strengths and limitations

There are a number of limitations to this study: (i) Details of smoking of parents and grandparents depend on parental self-report – however there is considerable information to indicate that adults are unlikely to lie about smoking habits, especially when using anonymised self-completion questionnaires;[32] here we have shown that the mean birth weight of the study mothers who had reported that their own mothers had smoked when they were *in utero* was 199g lower than that of those who had reported that their mother did not smoke at that time, which was about the expected order of difference if the mothers had reported accurately; (ii) although the amount the parents smoked was reported, there was no

For peer review only

estimate requested for the amount smoked by the grandmothers when pregnant with the study parent – this may have been associated with the outcome, but it is difficult to postulate how such effects might differ between the sexes of the study children; (iii) although the ALSPAC study is large, the numbers of women who smoked throughout pregnancy and for whom details are available on the grandmothers' smoking are reduced and consequently the statistical power is relatively low. Among the strengths of this study are the following: (a) it tested a prior hypothesis that early life exposures can have phenotypic effects down the paternal line with sex-specific outcomes; (b) the information on grandparental and parental smoking was collected prior to the birth of the study child, and consequently cannot have been biased by knowledge of fetal size; (c) birth length and head circumference were ascertained by trained measurers using standard techniques, as opposed to the generally inaccurate methods used in most delivery units; (d) IQ was measured using standard methodology by trained psychologists; (e) the study was based on a relatively large population sample, and results are therefore likely to be generalisable.

Meaning of the study

Our ~~previous~~parallel study of non-smoking mothers~~,~~ looked~~ing~~ at the effect of parental exposure *in utero*;~~,~~ we found the~~ir~~ sons were larger at birth (both in regard to birth weight and birth BMI) if the maternal grandmother had smoked in the pregnancy that resulted in the study mother. There was no discernible effect of paternal prenatal exposure on the study child's birth weight or BMI; however there was a slight *increase* in head circumference among the boys born to fathers who had been exposed *in utero* [mean difference +0.08 cm; 95% CI -0.03, +0.19].[8]

For peer review only

Attributing the smaller head circumference in boys of smoking mothers to the prenatal exposure of the father through his own mother's smoking, raises the question of possible mechanisms. How might the information be transmitted via his sperm or in some other way? As we noted in the introduction to this paper there is increasing evidence that exposures, especially in early life, can lead to enduring changes in the epigenome that, in turn, can modify gene expression. Whilst transgenerational epigenetic inheritance remains controversial, at least in humans,[33] the phenomenon of genomic imprinting establishes the principle that epigenetic marks such as DNA methylation placed in one generation can influence gene expression in the next. One such imprinted gene is the Insulin Growth Factor 2 (*IGF2*) which is expressed only from the paternally-derived chromosome 11, the maternal copy being epigenetically silenced. *IGF2* encodes an endocrine and autocrine/paracrine acting factor important in directing growth during prenatal development.[34,35] Maternal smoking has been shown to be associated with a 5% higher DNA methylation level at the *IGF2* DMR (differentially methylated region) in the newborn infant,[5] and interestingly in the context of our study, this methylation shift is specific to male offspring. Thus it is possible that the study father's *IGF2* DMR had been epigenetically modified (including in his fetal testes) by his mother's smoking throughout pregnancy. Furthermore it is plausible that this epigenetic state could be transmitted via his sperm to the study offspring. Imprinted gene regions tend to escape the usual widespread erasure of DNA methylation from the paternally-derived genome in the pre-implantation embryo soon after fertilisation.[36] In support of paternal effects generally, there is a report of hypomethylation at the *IGF2* DMR in umbilical cord blood being associated with paternal obesity suggesting a preconceptional impact of the obesity (and/or exposures related to it) on the reprogramming of imprint marks during spermatogenesis.[37]

For peer review only

Unanswered questions and further research

There are few studies on head circumference at birth. This is possibly because the measurements are generally inaccurate. Pilot studies before the start of the ALSPAC study had demonstrated that it was usually the student midwife who was given the task of measuring the circumference of the baby's head; she tended to have had little or no training and the measurements made were grossly inaccurate. For this study we only used measurements that were made by our own staff, after detailed training and with repeated validation over time. Detailed studies with accurate data to test the hypothesis raised by the results of this study are needed.

In conclusion

When the mother is a smoker, we found no effect of her own tobacco exposure *in utero* on the fetal growth of her children. However, when the mother is a smoker, paternal exposure *in utero* is associated with a reduced head circumference at birth and IQ at 8 years in sons, but not daughters. We had no prior hypothesis that head circumference would be associated, particularly among sons, so these results must be considered as hypothesis generating, and require testing in further longitudinal data sets.

Acknowledgements

We are extremely grateful to all the families who took part in this study, the midwives for their help in recruiting them, and the whole ALSPAC team, which includes interviewers, computer and laboratory technicians, clerical workers, research scientists, volunteers, managers, receptionists and nurses.

For peer review only

~~Authors' c~~Contribut<u>**ors**</u>**~~ions~~**: MP and JG had the idea; KN, SG and LLM carried out the statistical analyses; JG and MP wrote the first draft and all authors contributed to the final manuscript.

Funding: The UK Medical Research Council (MRC), the Wellcome Trust and the University of Bristol currently provide core support for ALSPAC. The statistical analyses for this paper were undertaken with funding from the Medical Research Council (grant no. G1100226). The funders had no role in study design, data collection, statistical analysis, decision to publish, or preparation of the manuscript.

Formatted: Font: (Default) Times New Roman, 12 pt, Bold

Competing interests: The authors have no competing interests.

Ethics approval

Ethical approval for the ALSPAC study was obtained from the ALSPAC Law and Ethics Committee and the three Avon-based Local Research Ethics Committees: Bristol and Weston Health Authority: E1808 Children of the Nineties: Avon Longitudinal Study of Pregnancy and Childhood (ALSPAC) (28th November 1989). Southmead Health Authority: 49/89 Children of the Nineties - "ALSPAC" (5th April 1990). Frenchay Health Authority: 90/8 Children of the Nineties. (28th June 1990). Written consent was obtained for all assays of biological samples. Ethics Committees considered voluntarily returned postal questionnaires as implied consent.

For peer review only

Data sharing: The ALSPAC data can be used by bona fide researchers. ALSPAC is committed to share data with bona fide researchers. See the study website for the conditions of use and access procedures: http://www.bristol.ac.uk/alspac/researchers/

For peer review only

22

References

1. Barker DJ. In utero programming of chronic disease. *Clin Sci* 1998;**95**:115-128.

2. Gluckman PD, Hanson MA, Buklijas T, et al. Epigenetic mechanisms that underpin metabolic and cardiovascular disease. *Nat Rev Endocrinol* 2009;**5**:401- 408. doi:10.1038/nrendo.2009.102

3. Relton C, Davey-Smith G, Ozanne S. Developmental Epigenetic Programming in Diabetes and Obesity. In: Jirtle R, Tyson E, eds. Environmental Epigenomics in Health and Disease; Epigenetics and Complex Diseases. Berlin Heidelberg: Springer 2013:235-253.

4. Breton CV, Byun HM, Wenten M, et al. Prenatal tobacco smoke exposure affects global and gene-specific DNA methylation. *Am J Respir Crit Care Med* 2009;**180**:462-467. Doi. 10.1164/rccm.200901-0135OC

5. Murphy SK, Adigun A, Huang Z, et al. Gender-specific methylation differences in relation to prenatal exposure to cigarette smoke. *Gene* 2012;**15**:36-43. doi.org/10.1016/j.gene.2011.11.062.

6. Joubert BR, Håberg SE, Nilsen RM, et al. 450K epigenome-wide scan identifies differential DNA methylation in newborns related to maternal smoking during pregnancy. *Environ Health Perspect* 2012;**120**:1425-1431. 10.1289/ehp.1205412.

7. Lee KKW, Pausova Z. Cigarette smoking and DNA methylation. *Front Genet* 2013;**4**:132. doi: 10.3389/fgene.2013.00132

8. Miller LL, Pembrey M, Davey Smith G, et al. Is the growth of the fetus of a non-smoking mother influenced by the smoking of either grandmother while pregnant? *PLoS ONE* 2014; 9(2): e86781. doi:10.1371/journal.pone.0086781~~In press~~.

For peer review only

9. Davey Smith G. Assessing intrauterine influences on offspring outcomes: Can epidemiological studies yield robust outcomes? *Basic Clin Pharmacol Toxicol* 2008;**102**:245-256. DOI: 10.1111/j.1742-7843.2007.00191.x

10. Macdonald-Wallis C, Tobias JH, Davey Smith G, et al. Parental smoking during pregnancy and offspring bone mass at age 10 years: findings from a prospective birth cohort. *Osteoporos Int* 2011; **22**:1809-1819. Doi: 10.1007/s00198-010-1415-y

11. Bygren LO, Kaati G, Edvinsson S. Longevity determined by ancestors' over nutrition during their slow growth period. *Acta Biotheor* 2001;**49**:53-59. Doi. 10.1023/A:1010241825519

12. Kaati G, Bygren LO, Edvinsson S. Cardiovascular and diabetes mortality determined by nutrition during parents' and grandparents' slow growth period. *Eur J Hum Genet* 2002;**10**:682-688. DOI:10.1038/sj.ejhg.5200859

13. Pembrey ME, Bygren LO, Kaati G et al. Sex-specific, male-line transgenerational responses in humans. *Eur J Hum Genet* 2006;**14**:159–166. doi:10.1038/sj.ejhg.5201538

14. Anway MD, Cupp AS, Uzumcu M, et al. Epigenetic transgenerational actions of endocrine disruptors and male fertility. *Science* 2005;**308**:1466-1469. DOI: 10.1126/science.1108190

15. Franklin TB, Russig H, Weiss IC et al. Epigenetic transmission of the impact of early stress across generations. *Biol Psychiatr* 2010;**68**:408-415.doi10.1016/j.biopsych.2010.05.036

16. Ng SF, Lin RC, Laybutt DR et al. Chronic high-fat diet in fathers programs β-cell dysfunction in female rat offspring. *Nature* 2010;**467**:963-966. doi:10.1038/nature09491

17. Zeybel M, Hardy T, Wong YK, et al. Multigenerational epigenetic adaptation of the hepatic wound-healing response. *Nat Med* 2012;**18**:1369–1377. doi:10.1038/nm.2893

For peer review only

18. Dunn GA, Bale TL. Maternal high-fat diet effects on third-generation female body size via the paternal lineage. *Endocrinol* 2011;**152**:2228-2236. Doi:10.1210/en.2010.1461

19. Burdge GC, Slater-Jefferies J, Torrens C, et al. Dietary protein restriction of pregnant rats in the F0 generation induces altered methylation of hepatic gene promoters in the adult male offspring in the F1 and F2 generations. *Br J Nutr* 2007;**97**:435-439. Doi 10.1017/S0007114507352392

20. Carone BR, Fauquier L, Habib N, et al. Paternally induced transgenerational environmental reprogramming of metabolic gene expression in mammals. *Cell* 2010;**143**:1084-1096. Doi:10.1016/j.cell.2010.12.008

21. Dias BG, Ressler KJ. Parental olfactory experience influences behavior and neural structure in subsequent generations. *Nat Neurosci* 2014;**17**:89-96. Doi: doi:10.1038/nn.3594

22. Daxinger L, Whitelaw E. Understanding transgenerational epigenetic inheritance via the gametes in mammals. *Nat Rev Genet* 2012;**31**:153-162. doi:10.1038/nrg3188

23. Misra DP, Astone N, Lynch CD. Maternal smoking and birth weight: interaction with parity and mother's own in utero exposure to smoking. *Epidemiol* 2005;**16**:288–293. doi: 10.1097/01.ede.0000158198.59544.cf

24. Golding J, Pembrey M, Jones R. ALSPAC–the Avon Longitudinal Study of Parents and Children. I. Study methodology. *Paediatr Perinatal Epidemiol* 2001;**15**:74–87. DOI: 10.1046/j.1365-3016.2001.00325.x

25. Boyd A, Golding J, Macleod J, et al. Cohort Profile: The 'Children of the 90s'–the index offspring of the Avon Longitudinal Study of Parents and Children. *Int J Epidemiol* 2012;**42**:111-127. doi. 10.1093/ije/dys064

26. ALSPAC web: http://www.bristol.ac.uk/alspac/researchers/resources-available/data-details/questionnaires/. Accessed 2 December 2013.

For peer review only

27. Cole TJ, Henson GL, Tremble JM, et al. Birthweight for length: ponderal index, body mass index or Benn index? *Ann Hum Biol* 1997;**24**:289–298. doi:10.1080/03014469700005032

28. Gale CR, O'Callaghan FJ, Bredow M, et al. The influence of head growth in fetal life, infancy, and childhood on intelligence at the ages of 4 and 8 years. *Pediatr* 2006;**118**:1486-1492. doi: 10.1542/peds.2005-2629

29. Wechsler D, Golombok S, Rust J. WISC-IIIUK Wechsler Intelligence Scale for Children. Sidcup, UK: Psychological Corp 1992.

30. Gunnell D, Miller LL, Rogers I, et al. Association of Insulin-like Growth Factor I and Insulin-like Growth Factor–Binding Protein-3 With Intelligence Quotient Among 8-to 9-Year-Old Children in the Avon Longitudinal Study of Parents and Children. *Pediatr* 2005; **116**:e681-e686. doi: 10.1542/peds.2004-2390

31. Dickens WT, Flynn JR. Heritability estimates versus large environmental effects: the IQ paradox resolved. *Psychol Rev* 2001;**108**:346. Doi:10.1037/0033-295X.108.2.346

32. Klebanoff MA, Levine RJ, Clemens JD, et al. Serum cotinine concentration and self-reported smoking during pregnancy. *Am J Epidemiol* 1998;**148**:259-262.

33. Grossniklaus U, Kelly B, Ferguson-Smith AC, et al. Transgenerational epigenetic inheritance: how important is it? *Nat Rev Genet* 2013;**14**:228-235. doi:10.1038/nrg3435

34. LeRoith D, Lowe WL. Growth factors. In: Melmed S, Conn PM, eds. Endocrinology: Basic and Clinical Principles, 2nd edition. Totowa, New Jersey: Humana Press 2005:85-91.

35. Demetriou C, Abu-Amero S, Thomas AC, et al. Paternally expressed, imprinted Insulin-1 like growth factor-2 in chorionic villi correlates significantly with birth weight. *PLoS ONE* 2014;doi:10.1371/journal.pone.0085454.

For peer review only

36. Seisenberger S, Peat JR, Hore TA, et al. Reprogramming DNA methylation in the mammalian life cycle: building and breaking epigenetic barriers. *Philos Trans R Soc Lond B Biol Sci* 2013;**368**: 20110330. doi: 10.1098/rstb.2011.0330.

37. Soubry A, Schildkraut JM, Murtha A, et al. Paternal obesity is associated with IGF2 hypomethylation in newborns: results from a Newborn Epigenetics Study (NEST) cohort. *BMC Med* 2013;29:doi:10.1186/1741-7015-11-29

For peer review only

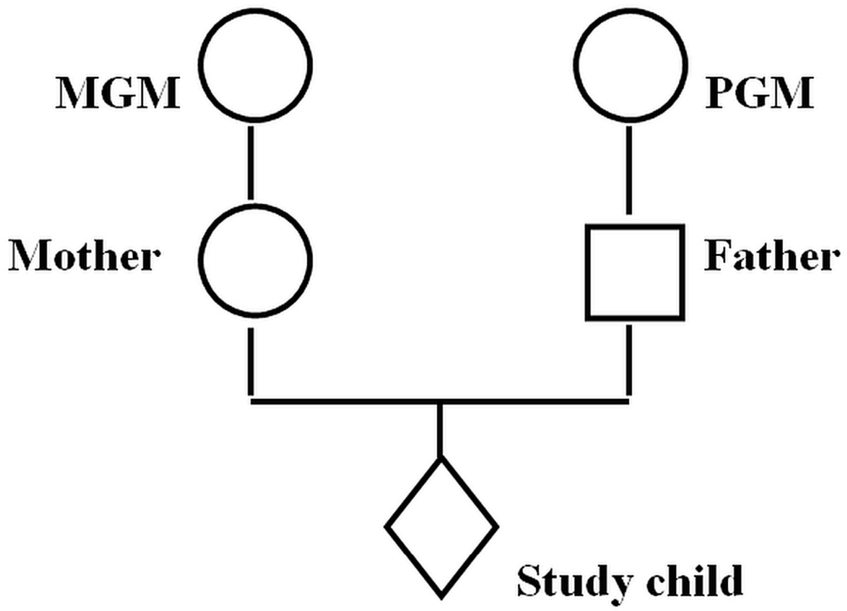

90x62mm (300 x 300 DPI)

view only

Supplementary Table 1. Mean difference (P value) [95% CI] in birth measurements of children born to smoking mothers, comparing those where the child's grandmother had smoked with those who had not when the child is the mother's firstborn

	MGM+ M+ v. MGM- M+		PGM+ M+ v. PGM- M+	
	Unadjusted	Adjusted[a]	Unadjusted	Adjusted[a]
**BIRTHWEIGHT (g)**				
Boy	-21 (0.64)	+5 (0.90)	-66 (0.22)	-51 (0.22)
	[-109, +67]	[-71, +80]	[-172, +40]	[-134, +31]
Girl	-79 (0.057)	-15 (0.69)	-86 (0.077)	-17 (0.69)
	[-160, +2]	[-92, +61]	[-181, +9]	[-101, +67]
**BIRTH LENGTH (cm x100)**				
Boy	+12 (0.58)	+22 (0.27)	-31 (0.22)	-33 (0.12)
	[-29, +52]	[-17, +61]	[-82, +19]	[-75, +09]
Girl	-26 (0.21)	+12 (0.58)	-34 (0.17)	-9 (0.71)
	[-66, +14]	[-31, +54]	[-84, +15]	[-57, +39]
**HEAD CIRCUMFERENCE (cm x100)**				
Boy	+6 (0.69)	+7 (0.62)	-37 (0.051)	**-34 (0.036)**
	[-23, +34]	[-21, +35]	[-74, +00]	**[-66, -2]**
Girl	-23 (0.068)	+1 (0.94)	-12 (0.46)	+6 (0.69)
	[-48, +2]	[-25, +27]	[-43, +20]	[-23, +35]
**BMI ((kg/m^2)x10)**				
Boy	-0.6 (0.59)	-0.1 (0.92)	-0.6 (0.65)	-1.1 (0.37)
	[-2.8, +1.6]	[-2.3, +2.1]	[-3.2, +2.0]	[-3.5, +1.3]
Girl	-2.6 (0.061)	-0.7 (0.64)	-2.6 (0.13)	-1.6 (0.35)
	[-5.2, +0.1]	[-3.6, +2.2]	[-6.0, +0.8]	[-5.0, +1.8]

[a]Adjusted for maternal education, partner smoked in pregnancy, gestation of study child and the amount the mother smoked

[N.B. the data for birth length and head circumference are given in cm x 100 so as to aid viewing]

For peer review only

Supplementary Table 2. Mean difference (P value) [95% CI] in birth measurements of children born to smoking mothers, comparing those where the child's grandmother had smoked with those who had not when the child is NOT the mother's firstborn

	MGM+ M+ v. MGM- M+		PGM+ M+ v. PGM- M+	
	Unadjusted	Adjusted[a]	Unadjusted	Adjusted[a]
**BIRTHWEIGHT (g)**				
Boy	-16 (0.67)	-52 (0.099)	-84 (0.061)	-51 (0.17)
	[-88, +57]	[-115, +10]	[-172, +4]	[-125, +22]
Girl	-54 (0.15)	+41 (0.22)	**-106 (0.027)**	-1 (0.98)
	[-127, +19]	[-108, +25]	**[-199, -12]**	[-77, +75]
**BIRTH LENGTH (cm x100)**				
Boy	+1 (0.96)	-15 (0.44)	**-52 (0.033)**	-26 (0.27)
	[-38, +40]	[-5, +24]	**[-101, -4]**	[-73, +21]
Girl	+1 (0.97)	+4 (0.82)	-5 (0.074)	+14 (0.53)
	[-39, +40]	[-32, +41]	[-95, +4]	[-30, +57]
**HEAD CIRCUMFERENCE (cm x100)**				
Boy	+4 (0.74)	-9 (0.47)	**-38 (0.016)**	**-35 (0.017)**
	[-21, +30]	[-35, +16]	**[-69, -7]**	**[-64, -6]**
Girl	-0 (0.98)	-2 (0.86)	-23 (0.12)	+11 (0.39)
	[-24, +23]	[-23, +19]	[-53, +6]	[-15, +37]
**BMI ((kg/m^2)x10)**				
Boy	-0.9 (0.45)	-1.2 (0.34)	-2.3 (0.11)	-1.1 (0.46)
	[-3.2, +1.4]	[-0.37, +1.3]	[-5.2, +0.5]	[-4.0, +1.8]
Girl	-1.1 (0.32)	-1.4 (0.21)	-2.1 (0.12)	+0.5 (0.87)
	[-3.4, +1.1]	[-3.7, +0.8]	[-4.8, +0.5]	[-2.0, +3.0]

[a]Adjusted for maternal education, partner smoked in pregnancy, gestation of study child and the amount the mother smoked

[N.B. the data for birth length and head circumference are given in cm x 100 so as to aid viewing]

For peer review only

STROBE Statement—checklist of items that should be included in reports of observational studies

	Item No	Recommendation
**Title and abstract**	1	(*a*) Indicate the study's design with a commonly used term in the title or the abstract
		(*b*) Provide in the abstract an informative and balanced summary of what was done and what was found
**Introduction**		
Background/rationale	2	Explain the scientific background and rationale for the investigation being reported
Objectives	3	State specific objectives, including any prespecified hypotheses
**Methods**		
Study design	4	Present key elements of study design early in the paper
Setting	5	Describe the setting, locations, and relevant dates, including periods of recruitment, exposure, follow-up, and data collection
Participants	6	(*a*) *Cohort study*—Give the eligibility criteria, and the sources and methods of selection of participants. Describe methods of follow-up<br>*Case-control study*—Give the eligibility criteria, and the sources and methods of case ascertainment and control selection. Give the rationale for the choice of cases and controls<br>*Cross-sectional study*—Give the eligibility criteria, and the sources and methods of selection of participants
		(*b*) *Cohort study*—For matched studies, give matching criteria and number of exposed and unexposed<br>*Case-control study*—For matched studies, give matching criteria and the number of controls per case
Variables	7	Clearly define all outcomes, exposures, predictors, potential confounders, and effect modifiers. Give diagnostic criteria, if applicable
Data sources/ measurement	8*	For each variable of interest, give sources of data and details of methods of assessment (measurement). Describe comparability of assessment methods if there is more than one group
Bias	9	Describe any efforts to address potential sources of bias
Study size	10	Explain how the study size was arrived at
Quantitative variables	11	Explain how quantitative variables were handled in the analyses. If applicable, describe which groupings were chosen and why
Statistical methods	12	(*a*) Describe all statistical methods, including those used to control for confounding
		(*b*) Describe any methods used to examine subgroups and interactions
		(*c*) Explain how missing data were addressed
		(*d*) *Cohort study*—If applicable, explain how loss to follow-up was addressed<br>*Case-control study*—If applicable, explain how matching of cases and controls was addressed<br>*Cross-sectional study*—If applicable, describe analytical methods taking account of sampling strategy
		(*e*) Describe any sensitivity analyses

Continued on next page

For peer review only

**Results**		
Participants	13*	(a) Report numbers of individuals at each stage of study—eg numbers potentially eligible, examined for eligibility, confirmed eligible, included in the study, completing follow-up, and analysed
		(b) Give reasons for non-participation at each stage
		(c) Consider use of a flow diagram
Descriptive data	14*	(a) Give characteristics of study participants (eg demographic, clinical, social) and information on exposures and potential confounders
		(b) Indicate number of participants with missing data for each variable of interest
		(c) *Cohort study*—Summarise follow-up time (eg, average and total amount)
Outcome data	15*	*Cohort study*—Report numbers of outcome events or summary measures over time
		Case-control study—Report numbers in each exposure category, or summary measures of exposure
		Cross-sectional study—Report numbers of outcome events or summary measures
Main results	16	(*a*) Give unadjusted estimates and, if applicable, confounder-adjusted estimates and their precision (eg, 95% confidence interval). Make clear which confounders were adjusted for and why they were included
		(*b*) Report category boundaries when continuous variables were categorized
		(*c*) If relevant, consider translating estimates of relative risk into absolute risk for a meaningful time period
Other analyses	17	Report other analyses done—eg analyses of subgroups and interactions, and sensitivity analyses
**Discussion**		
Key results	18	Summarise key results with reference to study objectives
Limitations	19	Discuss limitations of the study, taking into account sources of potential bias or imprecision. Discuss both direction and magnitude of any potential bias
Interpretation	20	Give a cautious overall interpretation of results considering objectives, limitations, multiplicity of analyses, results from similar studies, and other relevant evidence
Generalisability	21	Discuss the generalisability (external validity) of the study results
**Other information**		
Funding	22	Give the source of funding and the role of the funders for the present study and, if applicable, for the original study on which the present article is based

*Give information separately for cases and controls in case-control studies and, if applicable, for exposed and unexposed groups in cohort and cross-sectional studies.

Note: An Explanation and Elaboration article discusses each checklist item and gives methodological background and published examples of transparent reporting. The STROBE checklist is best used in conjunction with this article (freely available on the Web sites of PLoS Medicine at http://www.plosmedicine.org/, Annals of Internal Medicine at http://www.annals.org/, and Epidemiology at http://www.epidem.com/). Information on the STROBE Initiative is available at www.strobe-statement.org.

For peer review only
